# Supplementary material for: The effectiveness of prosocial policies: Gender differences arising from social norms
Source: PLoS One. 2022 Dec 27;17(12):e0275383. doi: 10.1371/journal.pone.0275383 (PMC9794096; doi:10.1371/journal.pone.0275383)

Supplementary Information on

"The effectiveness of prosocial policies: Gender differences arising from social norms"

Antonio Cabrales[1,2*], Ryan Kendall[1], Angel Sánchez[3,4]

1 Department of Economics, University College London, London, United Kingdom
2 Departamento de Economía, Universidad Carlos III de Madrid, Leganés, Madrid, Spain
3 Grupo Interdisciplinar de Sistemas Complejos (GISC), Departamento de Matemáticas, Universidad Carlos III de Madrid, Leganés, Madrid, Spain
4 Instituto de Biocomputación y Física de Sistemas Complejos (BIFI), Universidad de Zaragoza, Zaragoza, Spain

* antonio.cabrales@uc3m.es

A. Theory

As stated in the main text, our model depends on the following parameters: the speed of each strategy (S_F, S_S, and S_A), the idiosyncratic factor in accident probabilities (a_F, a_S, and a_A), and the exponent γ in the utility function. A general analysis of the model for any value of the parameters is beyond the scope of this paper, so from now on we will focus on a set of choices for the average speeds and accident probabilities that will be implemented in the experiment.[1] This set of parameters, in which γ_i is still free as we do not control risk preferences of the participants in the experiment, is as follows: [2]

In what follows, we will use the parameters $S_F = 2$, $S_S = 1$, $S_A = 0.5$; $a_F = 0.35$, $a_S = 0.3$, $a_A = 0$, $N = 10$. With this choice, we have

$$\begin{aligned} EU(F) &= 2^{\gamma}\left(1 - 0.35\left(\frac{9}{10}\left(2x_F + x_S + 0.5\left(1 - x_F - x_S\right)\right) + \frac{2}{10}\right)\right) \\ EU(S) &= \left(1 - 0.3\left(\frac{9}{10}\left(2x_F + x_S + 0.5\left(1 - x_F - x_A\right)\right) + \frac{1}{10}\right)\right) \\ EU(A) &= 0.5^{\gamma} \end{aligned}$$

We can now state our first result:

Proposition 1 *Suppose participants are heterogeneous in CRRA and their risk preferences γ_i are drawn from a distribution with CDF $G(.)$. The risk preferences are the private information of each individual, and the equilibrium concept is Bayes-Nash.*

1. *There are no beliefs about AS and no value of $\gamma_i \in (0,1)$ for which it is optimal to choose S.*
2. *If players $G(.)$ have positive mass for every $\gamma_i \in (0,1)$, there is no equilibrium where all types of all players only choose A or only F.*

[1] The choices are made for simplicity. Some of them are at present unrealistic, such as $a_A = 0$ but it allows us to test a situation where the policy benefit of controlling the proportion of F players is clear.

[2] Note that for our parameter values:

$$\begin{aligned} \frac{\partial\left(EU_i(F) - EU_i(S)\right)}{\partial AS_{-i}} &= \frac{N-1}{N}\left(a_S S_S^{\gamma_i} - a_F S_F^{\gamma_i}\right) = \frac{N-1}{N}\left(0.3 - 0.35 * 2^{\gamma}\right) < 0 \\ \frac{\partial\left(EU_i(S) - EU_i(A)\right)}{\partial AS_{-i}} &= \frac{N-1}{N}\left(a_A S_A^{\gamma_i} - a_S S_S^{\gamma_i}\right) = \frac{N-1}{N}\left(-0.3\right) < 0 \end{aligned}$$

To summarize an increase in AS_{-i} makes S more attractive with respect to F, and A more attractive with respect to S, which makes our game one of strategic substitutes.

Proof. For part 1 of the proposition to be true we need to show that action S is not a best response for all types for all possible beliefs about the average speed of others ($AS \in [0.5, 2]$) and for reasonable risk preferences ($\gamma \in (0,1)$). Suppose not, then there is some γ and AS for which

$$E(U(S)) > \max\{E(U(A)), E(U(F))\}.$$

$$\begin{aligned}
2^{\gamma}\left(1-0.35\left(\frac{9}{10}AS+\frac{2}{10}\right)\right) &< 1-0.3\left(\frac{9}{10}AS+\frac{1}{10}\right) \\
0.5^{\gamma} &= \frac{1}{2^{\gamma}} < \left(1-0.3\left(\frac{9}{10}AS+\frac{1}{10}\right)\right) \Leftrightarrow \frac{1}{\left(1-0.3\left(\frac{9}{10}AS+\frac{1}{10}\right)\right)} < 2^{\gamma} \\
\frac{\left(1-0.35\left(\frac{9}{10}AS+\frac{2}{10}\right)\right)}{\left(1-0.3\left(\frac{9}{10}AS+\frac{1}{10}\right)\right)} &< 2^{\gamma}\left(1-0.35\left(\frac{9}{10}AS+\frac{2}{10}\right)\right) < 1-0.3\left(\frac{9}{10}AS+\frac{1}{10}\right)
\end{aligned}$$

For such values to exist, it is necessary that

$$1 < \frac{\left(1-0.3\left(\frac{9}{10}AS+\frac{1}{10}\right)\right)^2}{\left(1-0.35\left(\frac{9}{10}AS+\frac{2}{10}\right)\right)}$$

The derivative of $\frac{\left(1-0.3\left(\frac{9}{10}AS+\frac{1}{10}\right)\right)^2}{\left(1-0.35\left(\frac{9}{10}AS+\frac{2}{10}\right)\right)}$ with respect to AS is

$$\begin{aligned}
&\frac{-0.6\frac{9}{10}\left(1-0.3\left(\frac{9}{10}AS+\frac{1}{10}\right)\right)\left(1-0.35\left(\frac{9}{10}AS+\frac{2}{10}\right)\right)+0.35\frac{9}{10}\left(1-0.3\left(\frac{9}{10}AS+\frac{1}{10}\right)\right)^2}{\left(1-0.35\left(\frac{9}{10}AS+\frac{2}{10}\right)\right)^2} \\
= &\left(1-0.3\left(\frac{9}{10}AS+\frac{1}{10}\right)\right)\frac{-0.6\frac{9}{10}\left(1-0.35\left(\frac{9}{10}AS+\frac{2}{10}\right)\right)+0.35\frac{9}{10}\left(1-0.3\left(\frac{9}{10}AS+\frac{1}{10}\right)\right)}{\left(1-0.35\left(\frac{9}{10}AS+\frac{2}{10}\right)\right)^2} \\
= &\left(1-0.3\left(\frac{9}{10}AS+\frac{1}{10}\right)\right)\frac{0.085\,05AS-0.196\,65}{\left(1-0.35\left(\frac{9}{10}AS+\frac{2}{10}\right)\right)^2} < 0
\end{aligned}$$

thus $\frac{\left(1-0.3\left(\frac{9}{10}AS+\frac{1}{10}\right)\right)^2}{\left(1-0.35\left(\frac{9}{10}AS+\frac{2}{10}\right)\right)} < 1$ for $AS \in [0.5, 2]$.

For part 2, notice that if all other players choose F, then it must be the case that for all $\gamma \in (0,1)$

$$\begin{aligned}
2^{\gamma}\left(1-0.35\cdot\left(\frac{9}{10}2+\frac{2}{10}\right)\right) &= 0.3\cdot 2^{\gamma} \geq 0.5^{\gamma} \\
0.3 &\geq 0.25^{\gamma}. \qquad (1)
\end{aligned}$$

Since inequality 1 is only true for $\gamma \geq \frac{-\ln 0.3}{-\ln 0.25} \simeq 0.868\,48$, then there is a contradiction.

If all other players choose A, then it must be the case that for all $\gamma \in (0,1)$

$$\begin{aligned}
2^{\gamma}\left(1-0.35\left(\frac{9}{10}0.5+\frac{2}{10}\right)\right) &= 0.7725\cdot 2^{\gamma} \leq 0.5^{\gamma} \\
0.825 &\leq 0.25^{\gamma}. \qquad (2)
\end{aligned}$$

Again, since 2 is only true for $\gamma \leq \frac{-\ln 0.7725}{-\ln 0.25} \simeq 0.186\,20$, we have a contradiction. ■

As stated in the main text, this proposition 1 leads to our first two hypotheses.

Hypothesis 1 *The proportion of players choosing S will be lower than those choosing A and F.*

Hypothesis 2 *Participants in the experiment will never completely coordinate on choosing A or F.*

As for the three treatments considered, let us recall that the utility of strategy F which can be written as:

$$EU_i(F) = ((1-q)\,U_i(S_F) + qU_i(S_F - C))\left(1 - a_F\left(\frac{N-1}{N}AS^C_{-i} + \frac{1}{N}S_F\right)\right).$$

We can establish the following proposition with resulting hypothesis.

Proposition 2 *A policy using monetary Exogenous, or Endogenou punishment, or Framing will decrease the proportion of players choosing F and the value of AS.*

Proof. Denote AS^C_{-i} as the equilibrium belief of player i about the average speed of other players under the punishment system C, q. Similarly, denote AS_{-i} as the belief of player i about the average speed of the other players without the system C, q. Proposition 1 states that the equilibrium will consist of a mixture of F and A players (S will never be chosen). The proportions of players choosing actions F and A are determined by the proportion of players that strictly prefer one action over the other. Since all $\gamma_i \in (0,1)$ are possible, there will be a type i player who is indifferent between the two actions.

$$U_i\left(S_F\right)\left(1-a_F\left(\frac{N-1}{N}AS_{-i}+\frac{1}{N}S_F\right)\right)=U_i\left(S_A\right)\left(1-a_A\left(\frac{N-1}{N}AS_{-i}+\frac{1}{N}S_A\right)\right).$$

With the punishment according to the C, q system, it must be that case that

$$\left(\left(1-q\right)U_i\left(S_F\right)+qU_i\left(S_F-C\right)\right)\left(1-a_F\left(\frac{N-1}{N}AS^C_{-i}+\frac{1}{N}S_F\right)\right)=$$

$$=U_i\left(S_A\right)\left(1-a_A\left(\frac{N-1}{N}AS^C_{-i}+\frac{1}{N}S_A\right)\right).$$

In a system without punishment, the indifferent type is such that

$$\frac{U_i\left(S_F\right)}{U_i\left(S_A\right)}=\frac{1-a_A\left(\frac{N-1}{N}AS_{-i}+\frac{1}{N}S_A\right)}{1-a_F\left(\frac{N-1}{N}AS_{-i}+\frac{1}{N}S_F\right)}. \tag{3}$$

In a system with punishment, for the indifferent type

$$\frac{\left(\left(1-q\right)U_i\left(S_F\right)+qU_i\left(\left(S_F-C\right)\right)\right)}{U_i\left(S_A\right)}=\frac{1-a_A\left(\frac{N-1}{N}AS^C_{-i}+\frac{1}{N}S_A\right)}{1-a_F\left(\frac{N-1}{N}AS^C_{-i}+\frac{1}{N}S_F\right)}. \tag{4}$$

When comparing the left side of equations 3 and 4, it is clear that

$$\frac{U_i\left(S_F\right)}{U_i\left(S_A\right)}>\frac{\left(\left(1-q\right)U_i\left(S_F\right)+qU_i\left(\left(S_F-C\right)\right)\right)}{U_i\left(S_A\right)}.$$

This means that the following must be true:

$$\frac{1-a_A\left(\frac{N-1}{N}AS_{-i}+\frac{1}{N}S_A\right)}{1-a_F\left(\frac{N-1}{N}AS_{-i}+\frac{1}{N}S_F\right)}>\frac{1-a_A\left(\frac{N-1}{N}AS^C_{-i}+\frac{1}{N}S_A\right)}{1-a_F\left(\frac{N-1}{N}AS^C_{-i}+\frac{1}{N}S_F\right)}$$

This immediately implies the following comparison between the Average Speeds across the two systems.

$$\left(1-a_F\left(\frac{N-1}{N}AS^C_{-i}+\frac{1}{N}S_F\right)\right)\left(1-a_A\left(\frac{N-1}{N}AS_{-i}+\frac{1}{N}S_A\right)\right)>$$

$$>\left(1-a_A\left(\frac{N-1}{N}AS^C_{-i}+\frac{1}{N}S_A\right)\right)\left(1-a_F\left(\frac{N-1}{N}AS_{-i}+\frac{1}{N}S_F\right)\right)>$$

$$-a_F\left(\frac{N-1}{N}AS^C_{-i}+\frac{1}{N}S_F\right)-a_A\left(\frac{N-1}{N}AS_{-i}+\frac{1}{N}S_A\right)>$$

$$>-a_A\left(\frac{N-1}{N}AS^C_{-i}+\frac{1}{N}S_A\right)-a_F\left(\frac{N-1}{N}AS_{-i}+\frac{1}{N}S_F\right)\left(a_F-a_A\right)AS_{-i}>$$

$$>\left(a_F-a_A\right)AS^C_{-i}AS_{-i}>$$

$$>AS^C_{-i}$$

Note that $AS = x_F S_F + x_S S_S + x_A S_A$. Proposition 1 states that x_S is zero without C, q, which means that it must be the case that for $AS_i > AS^C_i$ to be true, we must have that $x^P_F < x_F$. ■

Hypothesis 3 *The proportion of players choosing F will be lower in Exogenous, Endogeno or Framing than in Control.*

Up to now we have assumed that individuals differ only in their degree of risk aversion. But there is evidence that different individuals have different reactions to fines and social punishment. They may also have different beliefs about how others react to those interventions. Let us assume that there is an individual "sensitivity" to fines, which we denote α_i and let us also denote by AS_i^{P-i} the anticipation by player i of the reaction by other players when a fine is introduced. Then the utility can be written as:

$$EU(F) = ((1-p)\,U_i\,(S_F) + pU_i\,(S_F - \alpha_i P))\left(1 - a_F\left(\frac{N-1}{N}AS_i^{P_{-i}} + \frac{1}{N}S_F\right)\right).$$

Proposition 3 *A policy using monetary Exogenous, or Endogenous punishment, or Framing will decrease the proportion of players choosing F for individuals with high α_i . It will increase the proportion of players choosing F for those with low α_i who expect lower AS_i^{P-i}.*

Proof.

Denote AS_{-i}^P as the equilibrium belief of player i about the average speed of other players under the punishment system P, q. Similarly, denote AS_{-i} as the belief of player i about the average speed of the other players without the system P, q. Proposition 1 states that the equilibrium will consist of a mixture of F and A players (S will never be chosen). The proportions of players choosing actions F and A are determined by the proportion of players that strictly prefer one action over the other. Since all $\gamma_i \in (0,1)$ are possible, there will be a type i player who is indifferent between the two actions.

$$U_i\,(S_F)\left(1 - a_F\left(\frac{N-1}{N}AS_{-i} + \frac{1}{N}S_F\right)\right) = U_i\,(S_A)\left(1 - a_A\left(\frac{N-1}{N}AS_{-i} + \frac{1}{N}S_A\right)\right).$$

With the punishment according to the C, q system, it must be that case that

$$((1-q)\,U_i\,(S_F) + qU_i\,(S_F - \alpha_i C))\left(1 - a_F\left(\frac{N-1}{N}AS_{-i}^P + \frac{1}{N}S_F\right)\right) = U_i\,(S_A)\left(1 - a_A\left(\frac{N-1}{N}AS_{-i}^P + \frac{1}{N}S_A\right)\right).$$

For values of α_i close to 1, it is clear taht the argument is exactly as in the proof of proposition 2. On the other hand, if α_i is close to zero

$$U_i\,(S_F)\left(1 - a_F\left(\frac{N-1}{N}AS_{-i}^P + \frac{1}{N}S_F\right)\right) = U_i\,(S_A)\left(1 - a_A\left(\frac{N-1}{N}AS_{-i}^P + \frac{1}{N}S_A\right)\right).$$

In a system without punishment, the indifferent type is such that

$$\frac{U_i\,(S_F)}{U_i\,(S_A)} = \frac{1 - a_A\left(\frac{N-1}{N}AS_{-i} + \frac{1}{N}S_A\right)}{1 - a_F\left(\frac{N-1}{N}AS_{-i} + \frac{1}{N}S_F\right)}. \tag{5}$$

In a system with punishment, for the indifferent type

$$\frac{U_i\,(S_F)}{U_i\,(S_A)} \simeq \frac{((1-q)\,U_i\,(S_F) + qU_i\,(S_F - \alpha_i C))}{U_i\,(S_A)} = \frac{1 - a_A\left(\frac{N-1}{N}AS_{-i}^P + \frac{1}{N}S_A\right)}{1 - a_F\left(\frac{N-1}{N}AS_{-i}^P + \frac{1}{N}S_F\right)}. \tag{6}$$

For the people who believe that $AS_{-i}^P < AS_{-i}$ the right hand side of equation 6 now lowers with respect to that of 6 and this implies that the indifferent type will shift now in favor of strategy F, and there will be more mass of players who prefer F. ■

B. Participants statistics and Balance Check

Table 1 shows the session characteristics divided by condition.

Table 1: Session statistics by policy condition

Condition	# of participants	Avg. time of day	Avg. # of rounds	# of choices
Control	80	12:48	21.6	1,735
Framing	77	12:15	18.9	1,457
Exogenous	84	12:33	20.4	1,703
Endogenous	85	12:48	21.8	1,854
Total	326	12:36	20.7	6,749

Table 2 compares the participant characteristics divided by condition. In addition to gender and risk preference, participants are asked their age, whether they had ever owned a driver's license ("License"), and whether they learned during the experiment ("Learning"). P-values are shown for differences (from *Control*) using either a chi-squared test across measures using proportions (Female, License, and Learning) or a Mann-Whitney test across measures with ordinal data (Risk and Age).

Table 2: Participants statistics by policy condition

Condition	Female (prop.)	Avg. Risk	Avg. Age	License (prop.)	Learning (prop.)
Control	.51	3.94	24.2	.65	.60
Framing	.47	4.03	24.4	.71	.56
	($p = .69$)	($p = .59$)	($p = .48$)	($p = .49$)	($p = .71$)
Exogenous	.57	4.40	24.4	.64	.62
	($p = .55$)	($p = .06$)	($p = .61$)	($p = 1.0$)	($p = .93$)
Endogenous	.54	3.92	23.8	.72	.67
	($p = .83$)	($p = .88$)	($p = .90$)	($p = .44$)	($p = .44$)
AnyPolicy	.53	4.12	24.2	.69	.62
	($p = .91$)	($p = .36$)	($p = .66$)	($p = .58$)	($p = .88$)

Tables 3 and 4 compare the participant characteristics divided by condition and by gender. P-values are shown for differences (from *Control*) using either a chi-squared test (License and Learning) or a Mann-Whitney test (Risk and Age), depending on the data.

Out of the 32 tests performed in Tables 3 and 4, only one is significantly different. Thus, the conditions are well-balanced across gender.

C. Full regression results

Table S1: Driving choice (relative to Fast)

	Slow (1a)	Auto (1b)	Slow (2a)	Auto (2b)
AnyPolicy	0.494	0.664	-	-
	(0.79)	(1.16)		
AnyPolicy*Female	0.456^{a}	0.770^{**}	-	-
	(1.82)	(3.02)		
Endogenous	-	-	0.853	0.876
			(1.20)	(1.53)
*Endogenous**Female	-	-	0.744^{*}	0.732^{*}
			(2.36)	(2.41)
Exogenous	-	-	0.695	1.166^{a}
			(0.92)	(1.90)
*Exogenous**Female	-	-	0.192	0.599^{a}
			(0.59)	(1.66)
Framing	-	-	-0.015	0.036
			(-0.02)	(0.05)
*Framing**Female	-	-	0.375	0.997^{*}
			(1.37)	(2.47)
Female	0.139	-0.200	0.180	-0.204
	(0.66)	(-1.05)	(0.90)	(-1.06)
Risk	0.141	-0.137	0.146	-0.137
	(0.95)	(-1.58)	(1.02)	(-1.57)
P.Fast	-1.261^{***}	-0.539^{*}	-1.261^{***}	-0.539^{*}
	(-6.04)	(-2.26)	(-6.05)	(-2.26)
P.Slow	0.215^{*}	1.098^{***}	0.215^{*}	1.100^{***}
	(2.01)	(3.81)	(2.01)	(3.80)
P.Auto	0.217	1.964^{***}	0.217	1.966^{***}
	(1.18)	(6.69)	(1.18)	(6.69)
Late	-0.088	0.069^{*}	-0.087	0.069^{*}
	(-0.94)	(2.19)	(-0.93)	(2.17)
AnyPolicy*Risk	-0.200	-0.101	-	-
	(-1.33)	(-1.02)		
AnyPolicy*P.Fast	0.180	-0.520^{a}	-	-
	(0.65)	(-1.84)		
AnyPolicy*P.Slow	0.725^{***}	-1.035^{**}	-	-
	(3.65)	(-3.07)		
AnyPolicy*P.Auto	-0.235	-0.607^{a}	-	-
	(-0.88)	(-1.83)		
AnyPolicy*Late	-0.074	-0.065	-	-
	(-0.62)	(-1.07)		
*Endogenous**Risk	-	-	-0.265	-0.115
			(-1.69)	(-1.23)
*Endogenous**P.Fast	-	-	-0.093	-0.621^{a}
			(-0.25)	(-1.89)
*Endogenous**P.Slow	-	-	0.498^{a}	-1.242^{**}
			(1.86)	(-3.11)
*Endogenous**P.Auto	-	-	-0.483^{a}	-0.768^{a}
			(-1.69)	(-1.92)
*Endogenous**Late	-	-	-0.041	-0.058
			(-0.29)	(-0.53)
*Exogenous**Risk	-	-	-0.207	-0.185
			(-1.22)	(-1.38)
*Exogenous**P.Fast	-	-	0.292	-0.725^{a}
			(0.74)	(-1.86)
*Exogenous**P.Slow	-	-	0.549	-1.098^{*}
			(1.57)	(-2.40)
*Exogenous**P.Auto	-	-	-0.398	-0.734^{*}
			(-0.92)	(-2.00)
*Exogenous**Late	-	-	-0.157	-0.032
			(-0.87)	(-0.32)
*Framing**Risk	-	-	-0.144	-0.017
			(-0.91)	(-0.16)
*Framing**P.Fast	-	-	0.350	-0.204
			(1.05)	(-0.61)
*Framing**P.Slow	-	-	1.133^{***}	-0.725^{a}
			(3.86)	(-1.89)
*Framing**P.Auto	-	-	0.233	-0.359
			(0.71)	(-0.91)
*Framing**Late	-	-	-0.055	-0.084
			(-0.41)	(-1.28)
Session Size	-0.015	0.024	0.012	0.028
	(-0.30)	(0.61)	(0.20)	(0.67)
PropFemale	0.530	-0.439	-0.468	-0.430
	(-1.04)	(-1.12)	(-0.87)	(-1.09)
Constant	-0.895	-0.423	-0.882	-0.473
	(-1.12)	(-0.67)	(-1.05)	(-0.72)
N	6749	6749	6749	6749
Pseudo-R^2	0.163		0.166	

t statistics in parentheses
^{a} $p < 0.10$, * $p < 0.05$, ** $p < 0.01$, *** $p < 0.001$

Table S2: Beliefs about the driving choices in the population

	Fast Belief (3a)	Slow Belief (3b)	Auto Belief (3c)	Fast Belief (4a)	Slow Belief (4b)	Auto Belief (4c)
AnyPolicy	-0.564	-3.266	3.830	-	-	-
	(-0.17)	(-1.01)	(0.98)			
AnyPolicy*Female	-2.697	-1.816	4.513*	-	-	-
	(-1.43)	(-1.28)	(2.31)			
Endogenous	-	-	-	-2.022	-0.356	2.377
				(-0.51)	(-0.12)	(0.54)
*Endogenous**Female	-	-	-	-4.636*	-1.185	5.821**
				(-2.39)	(-0.80)	(3.00)
Exogenous	-	-	-	5.408	-9.635*	4.227
				(1.29)	(-2.42)	(0.75)
*Exogenous**Female	-	-	-	-3.884	-0.879	4.763
				(-1.54)	(-0.42)	(1.50)
Framing	-	-	-	-2.326	-1.327	3.653
				(-0.61)	(-0.29)	(0.82)
*Framing**Female	-	-	-	0.261	-3.163*	2.902[a]
				(0.16)	(-2.31)	(1.72)
Female	1.053	2.256*	-3.309*	0.972	2.394*	-3.366*
	(0.68)	(2.17)	(-2.15)	(0.63)	(2.48)	(-2.25)
Risk	0.006	-0.397	0.391	0.009	-0.400	0.391
	(0.01)	(-0.92)	(0.81)	(0.02)	(-0.92)	(0.81)
P.Fast	6.688**	-10.180***	3.492	6.677**	-10.163***	3.486
	(3.16)	(-4.33)	(1.11)	(3.16)	(-4.31)	(1.10)
P.Slow	0.762	-4.037*	3.274	0.724	-3.976*	3.252
	(0.24)	(-2.08)	(1.01)	(0.23)	(-2.05)	(1.00)
P.Auto	2.661	-10.380***	7.720*	2.705	-10.451***	7.746*
	(1.29)	(-4.22)	(2.43)	(1.31)	(-4.24)	(2.43)
Late	0.420	-4.223***	3.803*	0.405	-4.200***	3.795*
	(0.29)	(-9.88)	(2.64)	(0.28)	(-9.53)	(2.63)
AnyPolicy*Risk	0.749	0.645	-1.394*	-	-	-
	(1.12)	(1.17)	(-2.10)			
AnyPolicy*P.Fast	-2.413	3.395	-0.982	-	-	-
	(-0.96)	(1.19)	(-0.27)			
AnyPolicy*P.Slow	-0.540	4.231	-3.691	-	-	-
	(-0.16)	(1.61)	(-0.97)			
AnyPolicy*P.Auto	-1.643	1.940	-0.297	-	-	-
	(-0.68)	(0.65)	(-0.08)			
AnyPolicy*Late	3.089[a]	-0.438	-2.651	-	-	-
	(1.75)	(-0.45)	(-1.67)			
*Endogenous**Risk	-	-	-	1.027	0.416	-1.443[a]
				(1.58)	(0.72)	(-1.88)
*Endogenous**P.Fast	-	-	-	-4.420	4.385	0.036
				(-1.56)	(1.30)	(0.01)
*Endogenous**P.Slow	-	-	-	-0.869	2.175	-1.307
				(-0.22)	(0.64)	(-0.29)
*Endogenous**P.Auto	-	-	-	-2.765	2.940	-0.175
				(-0.90)	(0.87)	(-0.04)
*Endogenous**Late	-	-	-	3.818[a]	-1.228	-2.590
				(1.83)	(-0.88)	(-1.44)
*Exogenous**Risk	-	-	-	-0.277	1.555*	-1.278
				(-0.39)	(2.74)	(-1.49)
*Exogenous**P.Fast	-	-	-	-0.772	4.480	-3.707
				(-0.22)	(1.08)	(-0.77)
*Exogenous**P.Slow	-	-	-	0.144	6.151	-6.295
				(0.04)	(1.66)	(-1.29)
*Exogenous**P.Auto	-	-	-	-2.893	3.390	-0.497
				(-0.95)	(0.78)	(-0.09)
*Exogenous**Late	-	-	-	4.274[a]	-1.632	-2.642
				(1.80)	(-1.03)	(-1.60)
*Framing**Risk	-	-	-	1.008	0.191	-1.199
				(1.18)	(0.23)	(-1.46)
*Framing**P.Fast	-	-	-	-1.930	0.951	0.979
				(-0.67)	(0.31)	(0.24)
*Framing**P.Slow	-	-	-	-0.119	4.210	-4.092
				(-0.03)	(1.19)	(-1.06)
*Framing**P.Auto	-	-	-	-0.067	0.003	0.064
				(-0.02)	(0.00)	(0.02)
*Framing**Late	-	-	-	1.328	1.333	-2.661
				(0.67)	(0.88)	(-1.32)
Session Size	0.005	-0.534	0.530	0.151	-0.762	0.611
	(0.01)	(-0.57)	(0.45)	(0.21)	(-0.78)	(0.52)
PropFemale	4.214	5.343	-9.557	4.018	5.551	-9.569
	(0.56)	(0.64)	(-0.91)	(0.52)	(0.67)	(-0.95)
Constant	37.19***	36.33***	26.48*	35.82***	38.50***	25.68*
	(5.15)	(4.01)	(2.41)	(4.98)	(4.16)	(2.30)
N	6749	6749	6749	6749	6749	6749
Adj.-R^2	0.057	0.119	0.086	0.080	0.138	0.091

t statistics in parentheses
[a] $p < 0.10$, * $p < 0.05$, ** $p < 0.01$, *** $p < 0.001$

Table S3: Driving choice (relative to Fast) - Response to first experienced fine

	Logit	*Multinomial Logit*	
	Non-Fast	Slow	Auto
	(7)	(8a)	(8b)
AfterFine	-0.510	-1.185	-0.295
	(-0.60)	(-1.16)	(-0.32)
AfterFine*Female	0.575	0.501	0.762[a]
	(1.58)	(1.40)	(1.68)
Female	0.125	0.393	-0.196
	(0.48)	(1.13)	(-0.64)
Risk	-0.084	-0.050	-0.119
	(-0.92)	(-0.53)	(-1.05)
P.Fast	-0.540*	-0.562*	-0.483
	(-2.33)	(-2.26)	(-1.42)
P.Slow	0.252	0.296	0.147
	(1.09)	(1.02)	(0.47)
P.Auto	0.445[a]	-0.385	1.120***
	(1.80)	(-1.01)	(3.64)
Late	-0.030	-0.197	0.146
	(-0.05)	(-0.73)	(1.02)
AfterFine*Risk	-0.005	0.061	-0.044
	(-0.05)	(0.64)	(-0.36)
AfterFine*P.Fast	-0.377	-0.333	-0.097
	(-0.90)	(-0.65)	(-0.18)
AfterFine*P.Slow	0.358	0.881*	-0.097
	(0.88)	(2.10)	(-0.17)
AfterFine*Late	-0.018	0.160	-0.194
	(-0.07)	(0.46)	(-0.77)
Session Size	0.077	-0.010	0.172[a]
	(1.47)	(-0.13)	(1.74)
PropFemale	-1.103*	-1.190[a]	-1.116
	(-2.39)	(-1.69)	(-1.29)
Constant	-0.110	0.141	-1.875
	(-0.14)	(0.14)	(-1.58)
N	2042	2042	2042
Pseudo-R^2	0.092	0.103	

t statistics in parentheses

[a] $p < 0.10$, * $p < 0.05$, ** $p < 0.01$, *** $p < 0.001$

Table 3: Participants statistics by policy condition - Female participants

Condition	Avg. Risk	Avg. Age	License (prop.)	Learning (prop.)
Control	4.05	24.1	.61	.68
Framing	3.97	24.0	.72	.53
	($p = .86$)	($p = .60$)	($p = .42$)	($p = .25$)
Exogenous	4.04	24.7	.71	.58
	($p = .93$)	($p = .55$)	($p = .45$)	($p = .45$)
Endogenous	3.87	24.1	.70	.72
	($p = .46$)	($p = .50$)	($p = .54$)	($p = .91$)
AnyPolicy	3.96	24.3	.71	.62
	($p = .84$)	($p = .46$)	($p = .33$)	($p = .55$)

Table 4: Participants statistics by policy condition - Male participants

Condition	Avg. Risk	Avg. Age	License (prop.)	Learning (prop.)
Control	3.82	24.2	.69	.51
Framing	4.07	24.7	.71	.59
	($p = .49$)	($p = .63$)	($p = 1.0$)	($p = .67$)
Exogenous	4.89	24.1	.56	.67
	($p = .01$)	($p = .86$)	($p = .32$)	($p = .26$)
Endogenous	3.97	23.5	.74	.62
	($p = .66$)	($p = .42$)	($p = .80$)	($p = .49$)
AnyPolicy	4.29	24.1	.67	.62
	($p = .15$)	($p = .96$)	($p = .97$)	($p = .32$)

D. Earnings

In each round, a participant makes a driving choice of either Fast, Slow, or Auto. Their driving choice and the driving choice of others will determine their earnings in that round, as well as the (random) realization of an accident.[3] The average earnings from these choices separated by condition and gender are shown in Table 5.

Table 5: Earnings from driving choice by condition and gender (£)

	Control	*Endogenous*	*Exogenous*	*Framing*	AnyPolicy
All participants	10.82	10.58	10.86	10.73	10.68
Male participants	10.85	11.47	11.96	12.07	11.79
Female participants	10.80	9.82	10.01	9.22	9.68
Male - Female	0.05	1.65	1.95	2.85	2.11
Diff (p-value)	.918	$< .001$	$< .001$	$< .001$	$< .001$

Figure 1 which shows these comparisons graphically:

When analyzing all participants (top row of Table 5), none of the policy conditions produce significantly different average earnings relative to *Control* (p> 0.480 using a two-sample t-test for all 3 pairwise comparisons). Furthermore, earnings from driving choices in *Control* are not different across

[3]Participants also earn money for accurate beliefs about others, which is analyzed in the next section.

Figure 1: Earnings from driving choice by condition and gender (£)

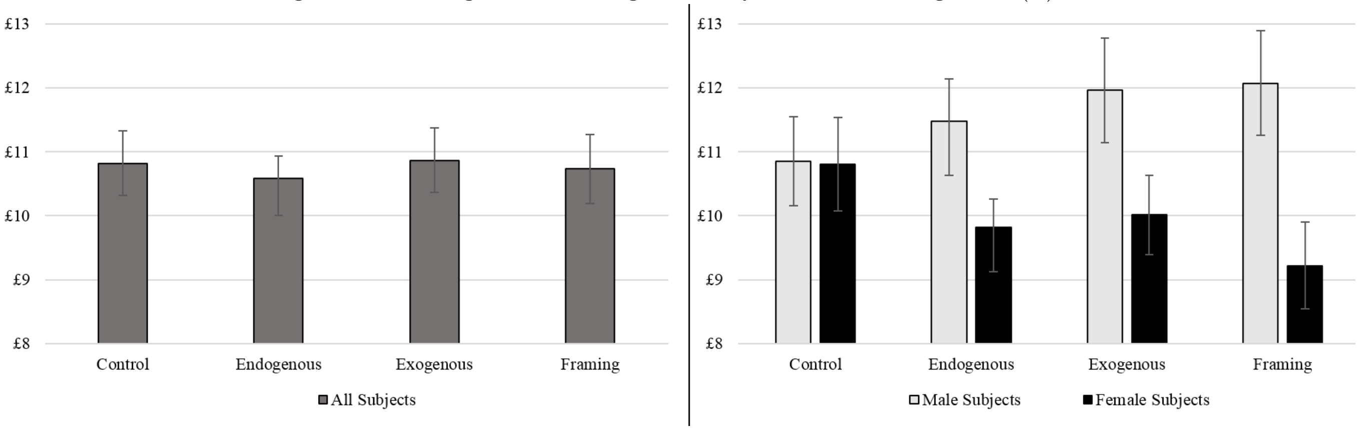

gender (p= 0.918). However, as with AS, separating by gender demonstrates a consistently different pattern: female participants in any policy condition earn significantly less.

We can further explore this relationship while controlling for independent variables collected as part of the experiment. We have independent dummy variables for each condition as well as a pooled AnyPolicy variable. In addition, we used their gender ("Female"=1 if the participant self-reports as female) and risk preferences (Holt and Laury 2002;"Risk" $\in [0, 10]$ where 10 is very risk-loving). We create dummy variables tracking the participant's driving choice in the immediately preceding round ("P.Fast" = 1 if the driving choice in the previous round was fast. Similarly variables for "P.Slow" and "P.Auto"). We also create a dummy variable tracking whether a choice is made in an early or late round of the session ("Late"= 1 in rounds after 10). Since the number of participants in a session varies between 8 and 12, we also control for "Session Size". We also control for the proportion of female participants in a session ("PropFemale" $\in [0.20, 0.80]$).[4]

Table 6 shows the results of 5 separate linear regressions used to identify the impact of these variables on a participant's earnings from the driving choice.[5]

Female participants earn significantly less within a policy (p = 0.001). This effect is mostly present in *Endogenous* and *Framing* (p = 0.067 and 0.057, respectively).[6] This effect is not present in *Control* (p = 0.660).

This analysis allows us to conclude the following: In *Control*, the earnings from driving choices do not differ by gender. In each policy condition, earnings from driving choices are lower for female participants when compared to male participants. Clearly, this is aligned with the result about the Average Speed discussed in the main text.

[4] We intentionally reduced the volatility of the gender mixture within each session as part of our experiment design. More specifically, we ensured there were at least two of each gender within every session.

[5] Standard errors are clustered at the session level. We use the 'reg' function with the 'vce' option in Stata.

[6] The effect is less pronounced in *Exogenous* (p = 0.128).

Table 6: Earnings

	Control	*Endogenous*	*Exogenous*	*Framing*	AnyPolicy
Female	-0.374	-1.310^{a}	-1.252	-2.120^{a}	-1.539***
	(-0.46)	(-2.16)	(-1.73)	(-2.28)	(-3.86)
Risk	-0.234	0.266	0.324	0.0630	0.212*
	(-0.78)	(1.29)	(1.85)	(0.45)	(2.11)
P.Fast	2.846	0.735	2.947^{a}	1.358	1.697*
	(1.42)	(0.73)	(2.07)	(1.19)	(2.42)
P.Slow	-0.491	-1.091^{a}	0.243	-1.108	-0.618
	(-0.23)	(-2.07)	(0.21)	(-0.70)	(-0.97)
P.Auto	-2.105	-2.463**	-0.661	-2.024	-1.728*
	(-1.25)	(-3.72)	(-0.51)	(-1.27)	(-2.57)
Late	-0.336	0.508	-0.332	0.355	0.161
	(-0.79)	(1.11)	(-0.81)	(1.17)	(0.67)
SessSize	-0.054	0.115*	0.110	0.221	0.071
	(-0.47)	(2.56)	(0.39)	(1.01)	(0.58)
PropFemale	0.829	1.069	1.189	-1.732	0.583
	(0.52)	(0.79)	(0.56)	(-0.51)	(0.49)
Constant	11.75***	8.717***	7.249	10.21**	9.380***
	(5.78)	(19.40)	(2.23)	(4.19)	(6.03)
N	1735	1854	1703	1457	5014
Pseudo-R^2	0.038	0.024	0.034	0.033	0.032

t statistics in parentheses

^{a} $p < 0.10$, * $p < 0.05$, ** $p < 0.01$, *** $p < 0.001$

E. Analysis of the contributions to punish Fast drivers

This section explores the factors that explain contribution decisions. Each of the 85 participants in *Endogenous* had the opportunity to contribute to the punishment fund in each round. As we mentioned before, participants who chose to contribute paid £1 which, in turn, would increase price of the fine paid by Fast drivers by £2.5. Out of a total of 1,854 opportunities to contribute, 209 contributions are made (11.3%). Slow drivers contribute 13.9% of the time, which is higher than Fast drivers or Auto drivers (10.4% and 10.5%, respectively). Table 7 groups the participants in *Endogenous* into contribution "types" based on the number of times they contributed. 48.2% of participants contributed to the fund at least once whereas three participants contributed in every round.[7] Table 7 also separates the classification by gender where no difference in behavior is observed (p = 0.556).

Table 7: Number of participants within each Contribution Type

Participants\Type	Never (0)	Seldom (1-2)	Moderate (3-8)	High (11-13)	Always (22-23)
All	44	23	10	5	3
	(51.8%)	(27.1%)	(11.8%)	(5.9%)	(3.5%)
Males	17	14	5	3	0
	(56.4%)	(25.6%)	(10.3%)	(7.7%)	(0%)
Female	20	15	6	2	3
	(47.8%)	(28.3%)	(13.0%)	(4.3%)	(6.5%)

Figure 2 displays the percentage of each driving choice separated by contribution types. Higher contribution types do not monotonically relate to any driving choice (which is also true when ignoring the "Always" contribution type).

[7] Interestingly, the three "Always" participants employed the same driving choice in every round they played. One participant chose Fast every round, one participant chose Slow every round, and one participant chose Auto every round. All three "Always" subjects were female students.

Figure 2: Participants choices by punishment types

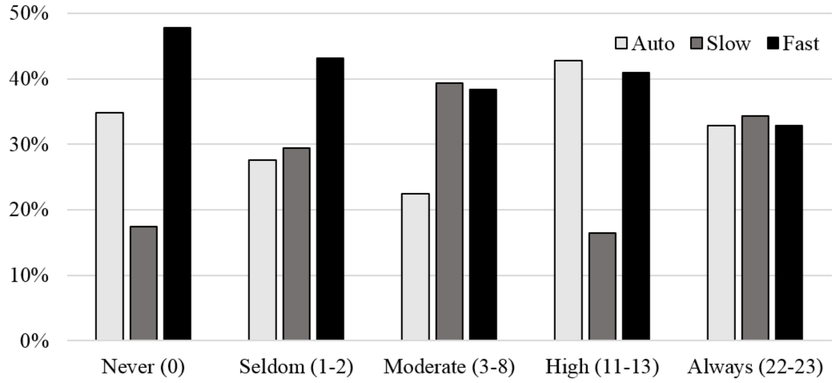

Female participants contribute to the punishment fund 13.5% of the time, which is higher than male participants (8.6%). However, these differences are based on only 8 sessions of *Endogenous* and a logit model which clusters at the session level shows this difference to be insignificant.

Table 8: Contribution choice

Female	0.422
	(0.66)
Risk	-0.157
	(-0.80)
P.Fast	-0.522
	(-1.53)
P.Slow	-0.213
	(-0.49)
P.Auto	-0.345
	(-1.15)
Late	-0.703***
	(-3.63)
Session Size	-0.238
	(-1.37)
PropFemale	1.034
	(0.67)
Constant	0.946
	(0.50)
N	1854
Pseudo-R^2	0.046

t statistics in parentheses

^{a} $p < 0.10$, * $p < 0.05$, ** $p < 0.01$, *** $p < 0.001$

The only significant predictor of contribution decisions is whether the participant is in the late rounds of the experiment. As shown in Figure 3, the proportion of participants contributing to the punishment fund declines over the duration of the experiment. With this one exception, our data suggest that the decision to contribute to the punishment fund is orthogonal to driving choice, gender, risk preference, and driving choices from the previous round.

Figure 3: Contribution decisions by round

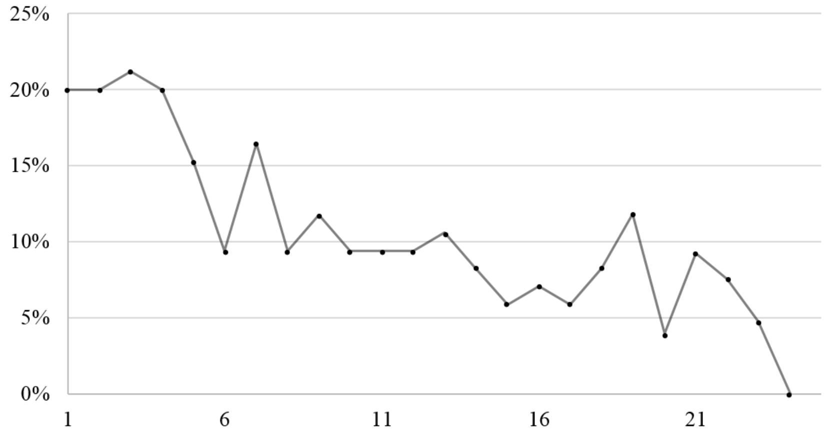

F. Full experimental instructions

Notes for this document. These are the instructions for an experiment with 4 treatment conditions – Control, Framing, Endogenous punishment, and Exogenous punishment. The text outlines the Control condition. Where appropriate, additions are shown for the Endogenous punishment and Exogenous punishment conditions. The Framing condition is the exact same as the Control condition with the exception that the words "Fast", "Auto", and "population" are replaced with "Reckless", "Safe", and "community".

----------------------Start of Instructions---------------------

Slide 1

Welcome to this experiment at UCL. Thank you for signing up.

You are about to participate in a study of decision-making, and you will be paid for your participation in cash. The amount you earn for participating in this experiment depends partly on your decisions, partly on the decisions by other subjects in the room, and partly on chance. There are several parts to this experiment, and your earnings will be totaled up and paid to you privately in the other room at the end of the experiment. This experiment is scheduled to last for 1.5 hours.

In order to use your data for research, you need to stay in the room for the entire experiment. However, your participation is voluntary and if you need to leave the room, you may do so at any time. If you choose to leave the room before the experiment is over, you will only be paid the £5 show-up fee and nothing that you have earned during the experiment.

Please turn off your mobile phone. It is important that you do not communicate with any other participants in the room during the experiment.

If you have a question about what you are being instructed to do in this experiment, please raise your hand and an experimenter will come over to your station. Please do not ask questions aloud. We encourage questions that help clarify how the experiment works. However, we cannot answer questions about how you should make choices in the experiment.

First, we will go through some instructions detailing what is expected of you during the experiment. This will include some examples and practice questions. You will not be paid according to these practice questions and the exact practice questions will never be asked as part of the decision periods of the experiment. After the instructions, you will participate in the decision rounds where you will be paid according to your choices. You will be reminded when the instructions and practice have concluded and the decision rounds are about to begin.

When you are ready, please click "Continue" to go to the instructions.

Slide 2

In this experiment, you have to choose the driving method you use to drive to work. You can choose to either drive "Fast", "Slow", or "Auto". These three methods differ in their earnings and their percent chance ("% chance") of being in a car accident.

(1) Choosing "Fast" represents a fast driving speed and a high % chance of being in an accident. This driving method has a high level of potential earnings along with a high % chance of being in an accident.

(2) Choosing "Slow" represents a slow driving speed with a low % chance of being in an accident. This driving method has a moderate level of potential earnings along with a moderate % chance of being in an accident.

(3) Choosing "Auto" represents a decision to let the car drive automatically. This driving method has a low level of potential earnings with a 0% chance of being in an accident.

Including yourself, there are 10 subjects in the room today. Your choice of a driving method affects the % chance that you are in an accident. If you choose to drive Fast or Slow, this % chance also depends on the choices of the other subjects in the room. When you have read this page, please click "Continue" to move onto the next instructions page.

Slide 3

Since there are 10 subjects in the room, the "Average Speed" is calculated by the following equation:

Average Speed = 2 x (# Fast drivers) /10 + 1 x (# Slow drivers) /10 + 0.5 x (# Auto drivers) /10

Intuitively, this equation means that each Fast driver increases the Average Speed the most (a factor of 2), each Slow driver increases the Average Speed a moderate amount (a factor of 1), and each Auto driver increases the Average Speed the least (a factor of 0.5).

The % chance that you are in an accident is determined by the Average Speed as well as your choice to drive either Fast, Slow, or Auto. If you are in an accident, no matter your driving choice, you earn £0.

If you choose to drive Fast, the % chance that you are in an accident is 0.35 x Average Speed

If you choose to drive Slow, the % chance that you are in an accident is 0.30 x Average Speed

If you choose to drive Auto, the % chance that you are in an accident is 0

Intuitively, this means that Fast drivers are always more likely to be in an accident than Slow drivers, Slow drivers are always more likely to be in an accident than Auto drivers, and Auto drivers are never in an accident.

If you are not in an accident, the amount of money you earn is determined by your choice to drive either Fast, Slow, or Auto.

If you choose to drive Fast and you are not in an accident, you will earn £28.

If you choose to drive Slow and you are not in an accident, you will earn £14.

If you choose to drive Auto, you are guaranteed not to be in an accident and you will earn £7.

Intuitively, this means that Fast drivers have the potential to earn the most, Slow drivers have the potential to earn the next most, and Auto drivers are guaranteed to earn £7.

[Italics only included in the exogenous punishment treatment]

Driving Fast also includes a 25% chance that you will be given a speeding fine of £4. This fine is only applied if you are not in an accident in that round. If you drive Fast, are not in an accident, but are given a fine, you will earn £24 (£28 - £4).

[Bold text only included in the endogenous punishment treatment]

Finally, every subject can choose whether or not to contribute £1 into the "speeding fine fund". Choosing to drive Fast includes a 25% chance that you will be given a speeding fine. The price of this fine is equal to 2.5 times the number of other drivers who contribute to the speeding fine fund. This fine is only applied to Fast drivers who are not in an accident. For example, consider a setting where two drivers contribute to the speeding fine fund. In this case, if you drive Fast, are not in an accident, but are given a fine, you will earn £23 (£28 - £(2*(2.5))).

Don't worry, you don't need to perfectly remember these equations. During the decision rounds, you will be given a tool that automatically calculates these equation for you. Click "Continue" to learn about this tool.

Slide 4

As part of the experiment, you will need to make a guess about the percentage of Fast, Slow, and Auto drivers in the room. You will use this "triangle" tool below to make this guess. As will be explained later, part of your earnings will be based on how accurately your guess matches the actual percentages of Fast, Slow, and Auto drivers. Also, the actual percentages of Fast, Slow, and Auto drivers in the room will determine the % chance that each driving method will have an accident. We will explain this over the next few slides. To activate the triangle tool, left-click anywhere inside of it. Please activate the triangle tool now.

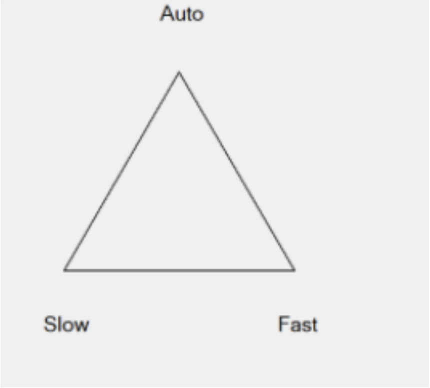

Slide 5

Good job! You can use the yellow "slider" to make a guess about the choices of the other participants in the room (the "population"). When you place the slider close to a point on the

triangle, you are guessing that many subjects in the room will make that choice. For example, if you wanted to show a guess that everyone in the room (including yourself) will choose Slow, then you would move the slider all the way to the bottom left of the triangle so that it is closest to the "Slow" point of the triangle. Please move the slider to show 100% of the population will choose Slow. Notice that your guess of the population includes your own choice. Then click "Continue".

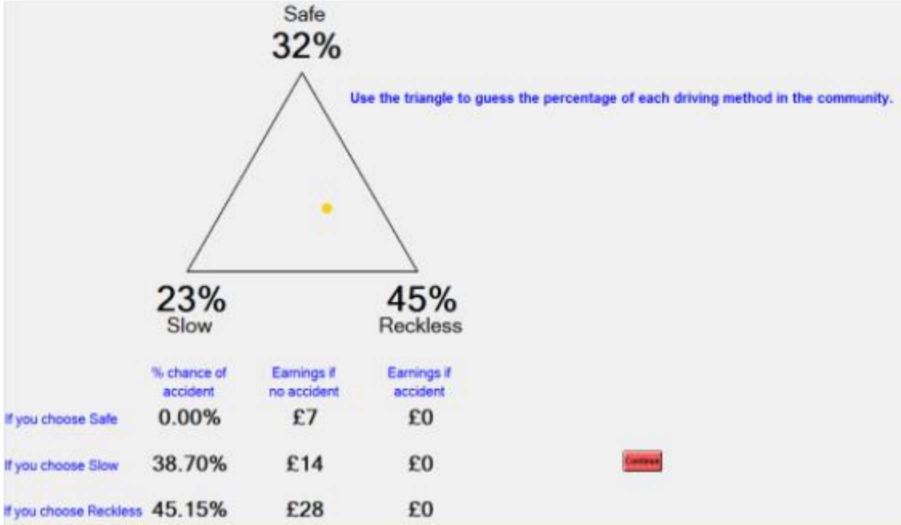

Slide 6

Great job! As you have noticed, when you move the slider, you change the percentage you are guessing is in each driving choice. Maybe, instead, you think that most subjects will choose Slow, but a few will choose Fast. Please move the slider to show 82% of the population will choose Slow and 18% will choose Fast. Then click "Continue".

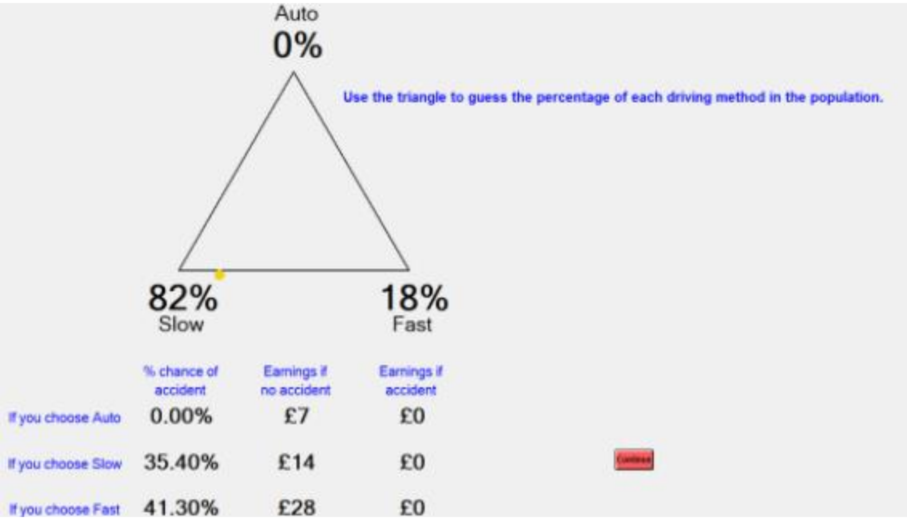

Slide 7

Please move the slider to show 40% of the population will choose Slow, 35% will choose Fast, and 25% will choose Auto. Then click "Continue".

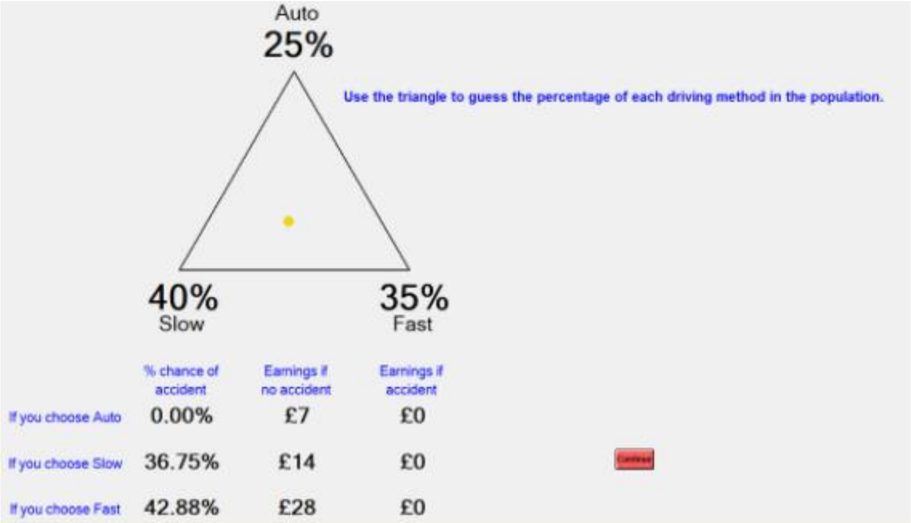

Slide 8

Great! You probably noticed that the % chance of an accident changes as you move around the slider. These percentages are using the equations from the earlier screens in order to automatically calculate the % chance of having an accident. Feel free to move the slider around to see how it affects these numbers. Then use the slider to answer the question below. Type in your % chance of being in an accident if you choose the Slow driving method and the population has the following distribution: 10% of choose Slow, 50% choose Fast, and 40% choose Auto. (do not include a percent sign)

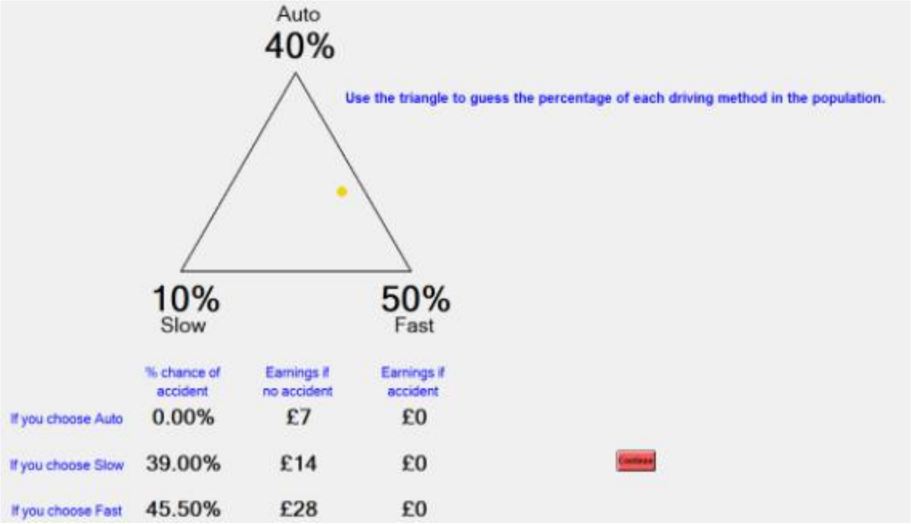

Slide 9

Your task today is to choose whether you want to drive Fast, Slow, or Auto. After you have used the triangle tool to make your guess about what driving methods you think that the people in the room will choose, you must choose one of these methods for yourself. In the experiment, you will make this choice by selecting one of the circles on the right hand side of the screen. In order to show an example, we locked the slider in the position it is at now. If that were your guess

about the population, your choice is the following: If you choose Fast, you have a 45.5% chance of earning £0 and a 54.5% chance of earning £28. If you choose Slow, you have a 39% chance of earning £0 and a 61% chance of earning £14. If you choose Auto, you have a 100% chance of earning £7. This is just a practice round, but think about what you might do here and make a choice. Then click "Submit".

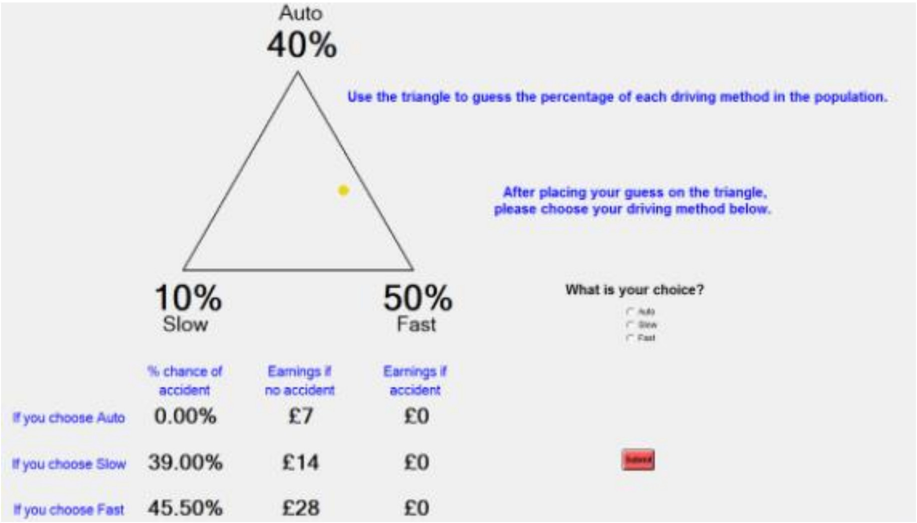

Slide 10

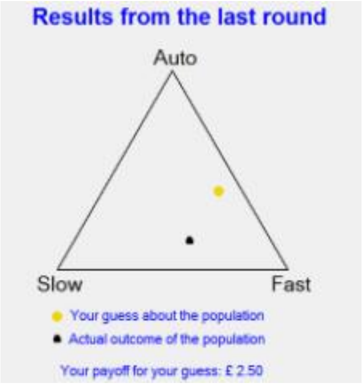

After you make your guess and your driving method choice, you will see a results screen that summarizes your earnings for the round. This screen will show the amount you earned based on your guess. As you would expect, you will earn more if your guess is closer to what the population actually did. The amount you earn from your guess is calculated by adding up the differences between your % guess of a driving method and the actual % of subjects who chose that driving method. In the example above. The guess of Fast, Slow, and Auto drivers is 50%, 10%, and 40%, respectively. The actual percentage of Fast, Slow, and Auto drivers is 50%, 35%, and 15%, respectively. So the Fast guess was 0 % points away from the actual population, the Slow guess was 25 % points away from the actual population, and the Auto guess was 25 % points away from the actual population. For your guess, you are paid £5 minus .05 times the number of % points your guess is away from the actual population. So, using this example, the guess was inaccurate by 50 % points (0+25+25) which would earn £2.50 (£5 - .05 x 50).

Intuitively, this means that you will earn £5 for a perfectly correct guess and that you will earn less with a less accurate guess. If your guess is too inaccurate, you will earn £0 for your guess. This will be calculated for you and displayed on the results screen in every round.

The main message from this slide is that you will earn more money the closer your guess is to the actual outcome. Please click "Continue" to move onto the next instructions.

Slide 11

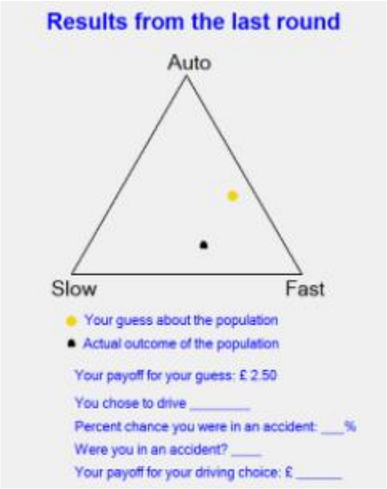

Your earnings from your driving method choice will also be shown on this screen. Your earnings will be based on your driving method choice as well as the actual driving methods chosen by the other participants in the room. Please note that the % chance that you are in an accident is computed according to the actual decisions of the subjects in the room (not according to your guess). Using the example above, your guess was that 40% will choose Auto, 10% will choose Slow, and 50% will choose Fast, but what actually happened was that 15% chose Auto, 35% chose Slow, and 50% chose Fast. These last percentages (represented by the black dot) are the ones that will be used to compute your % chance of being in an accident. If you choose either Slow or Fast, there will be a % chance that you will be in an accident and earn £0. This % chance will be displayed on this screen. Then, using this % chance, the computer will randomly determine whether or not you were in an accident. This outcome will be displayed.

[Italics only included in the exogenous punishment treatment]

If you chose to drive Fast, there is a 25% chance that you are fined £4. The computer will randomly determine whether or not you were fined. This outcome will be displayed.

[Bold text only included in the endogenous punishment treatment]

If you chose to drive Fast, there is a 25% chance that you are fined. The price of the fine equals 2.5 times the number of subjects who contributed to the speeding fine fund in that round. The computer will randomly determine whether or not you were fined. This outcome will be displayed.

Please click "Continue" to move onto the next instructions.

Slide 12

The experiment consists of a guess choice and a driving method choice made over many rounds. The experiment will last between 18 and 25. The exact number of rounds is randomly determined by the computer. Starting in Round 2, you will see a history bar at the bottom of the screen describing your choices and the outcome in the previous rounds. In every round, every subject in the room has the exact same options and payoffs that you have. Choices in previous rounds do not have any influence on following rounds (i.e., each round is independent). The conditions are identical for all of the rounds. We are now ready to begin the rounds that will determine your earnings in £. After you have completed all of the rounds, you will be shown a list displaying your guess earnings and driving method choice earnings in every round. From this list, the computer will randomly select one round to determine what you receive based on the accuracy of your guess. After that, the computer will randomly select another (different) round to determine what you receive based on your driving method choice. Because you will be paid based on the outcome of two of the upcoming rounds, it is important to take your time in each round and make choices that you are satisfied with.

If you have questions at any time during the experiment please raise your hand and an experimenter will come to assist you. Please click "Continue" to start the rounds.

----------------------End of Instructions---------------------

G. EScreen shots

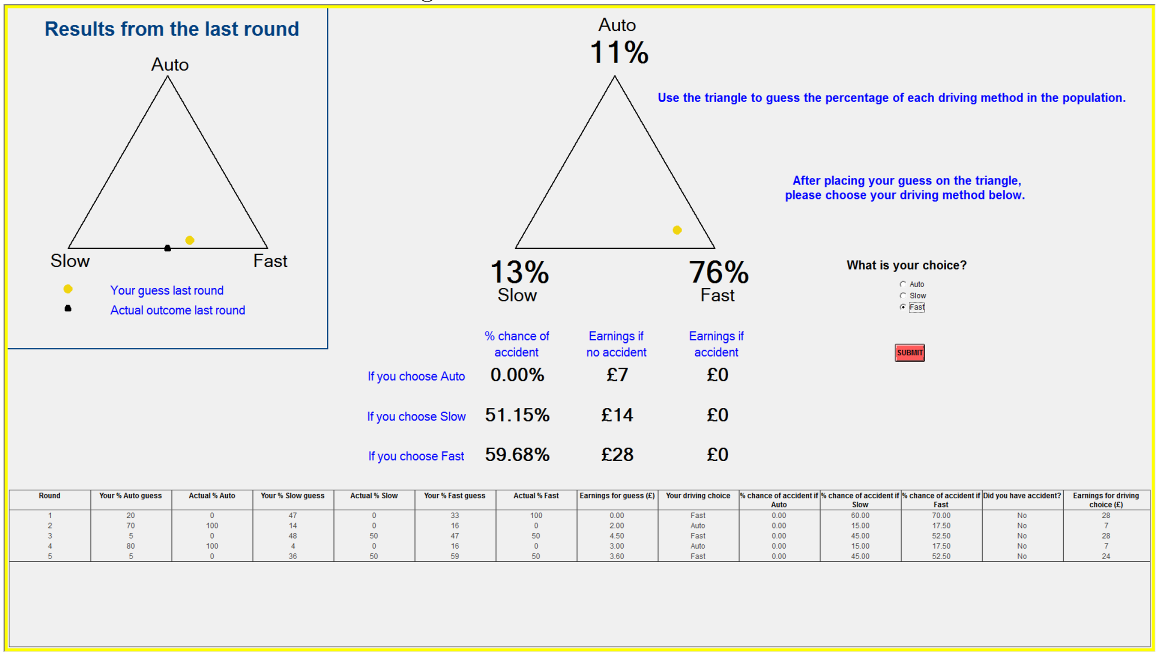

Figure 4: *Control* choice screen

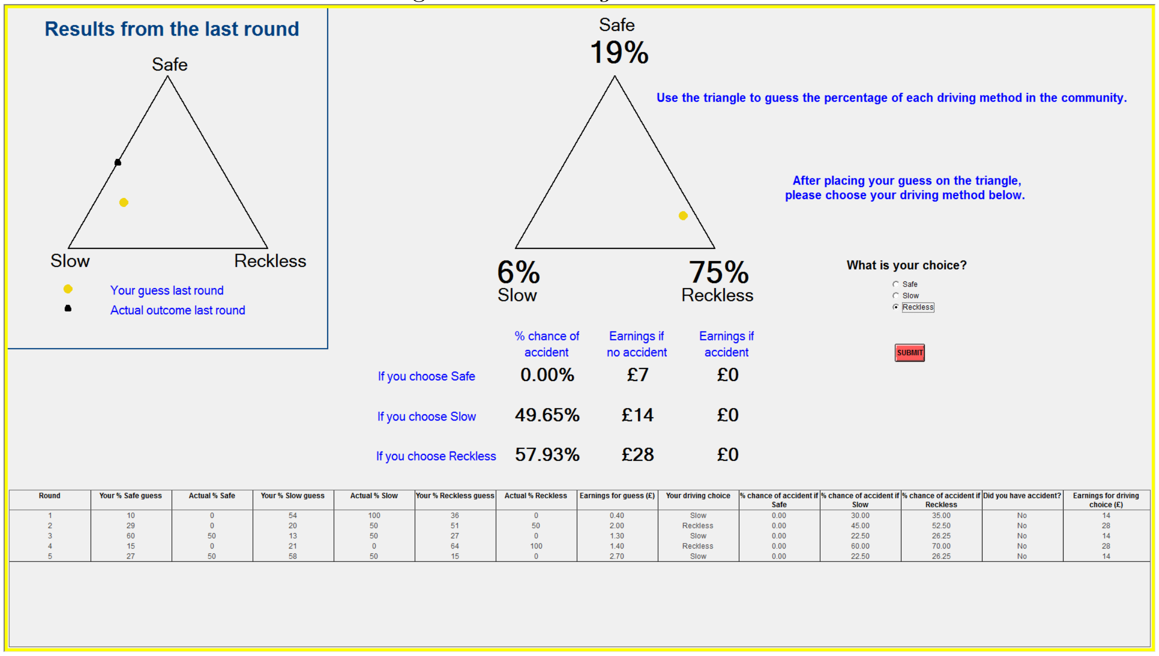

Figure 5: *Framing* choice screen

Figure 6: *Exogenous* punishment choice screen

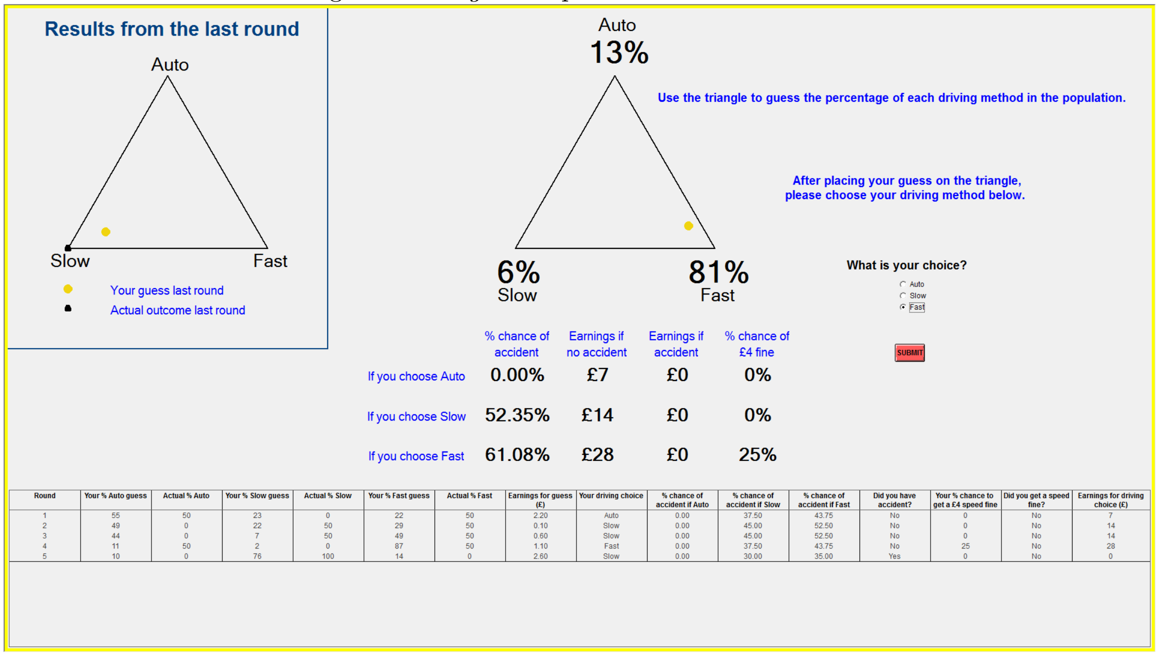

Figure 7: *Endogenous* punishment choice screen

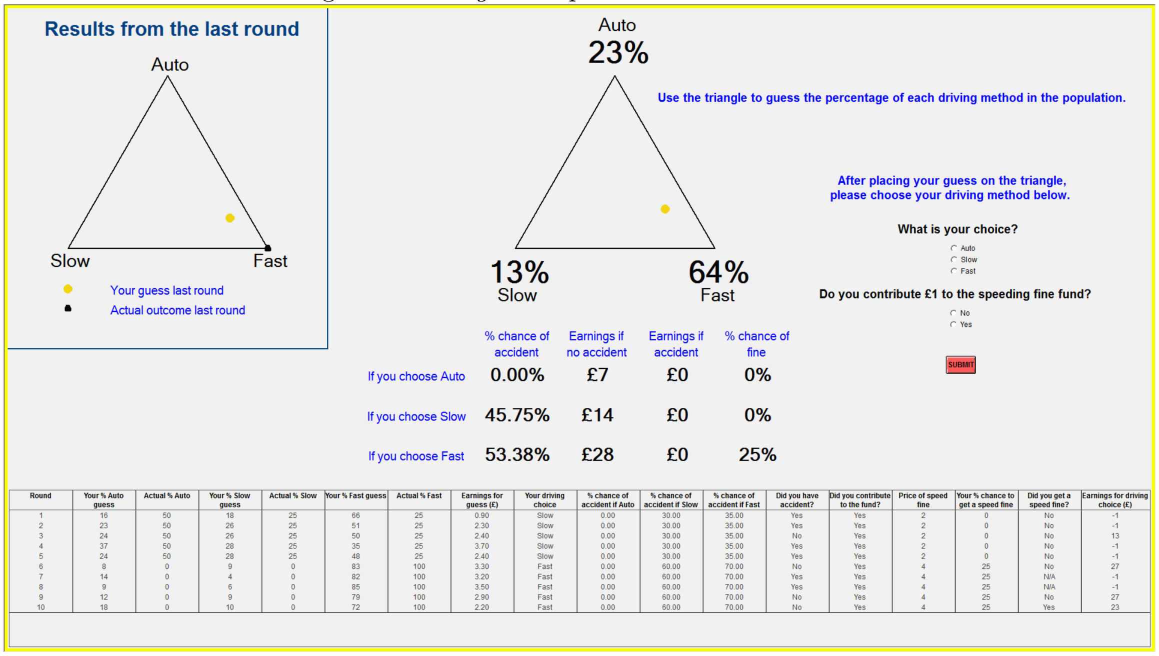

Figure 8: *Control* results screen

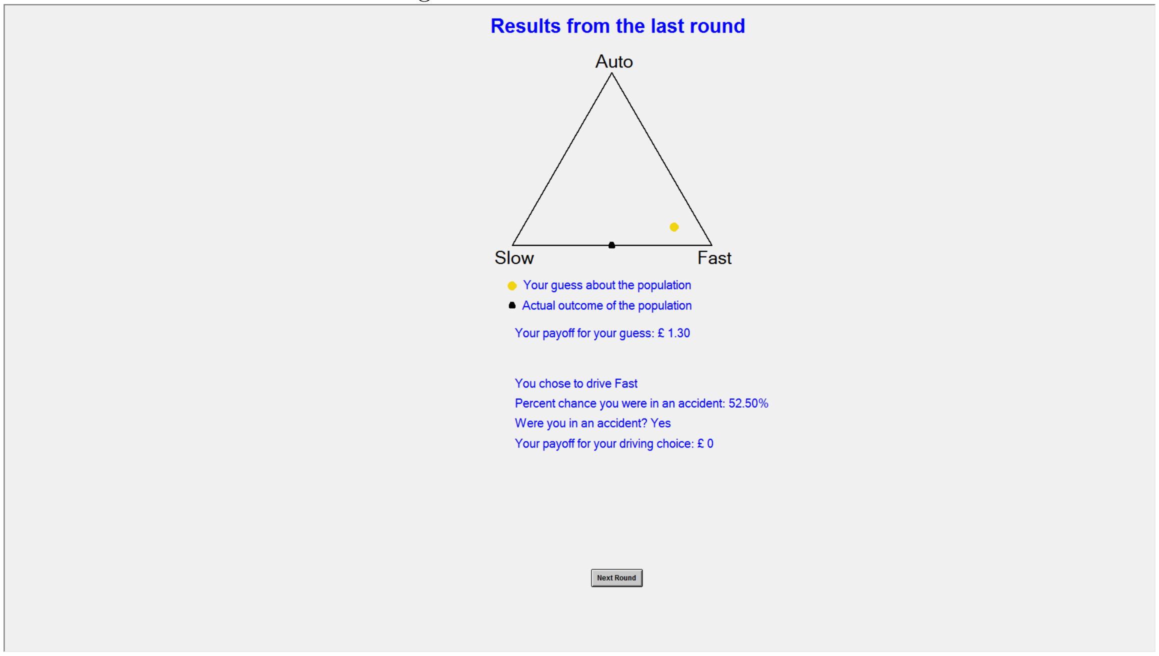

Figure 9: *Framing* results screen

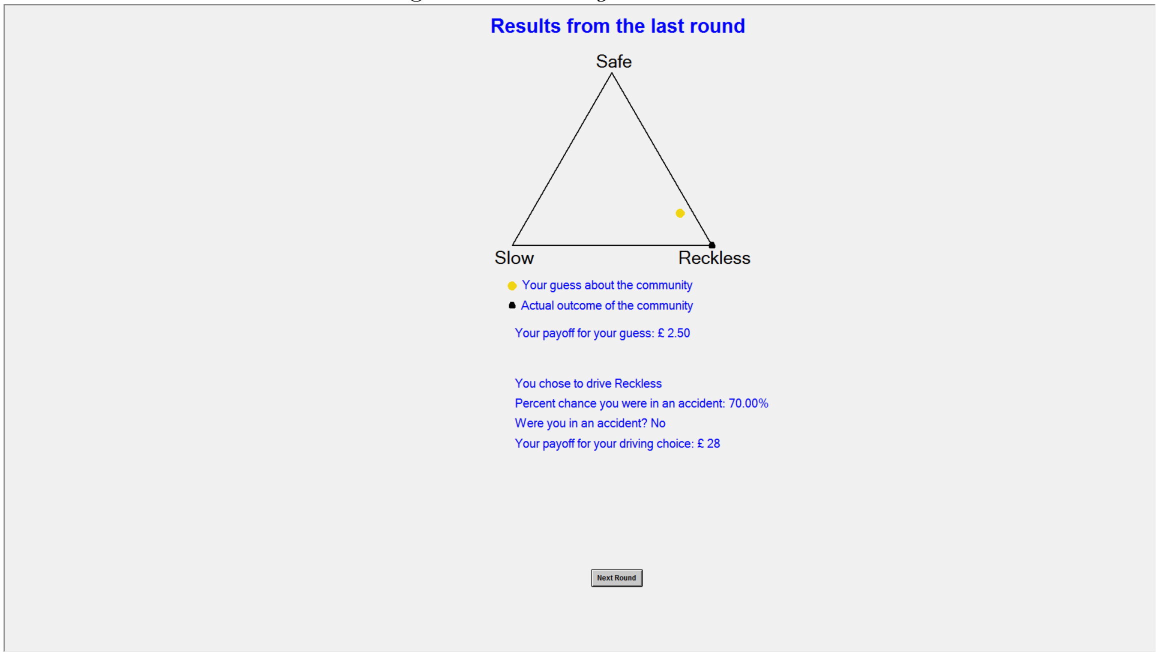

Figure 10: *Exogenous* punishment results screen

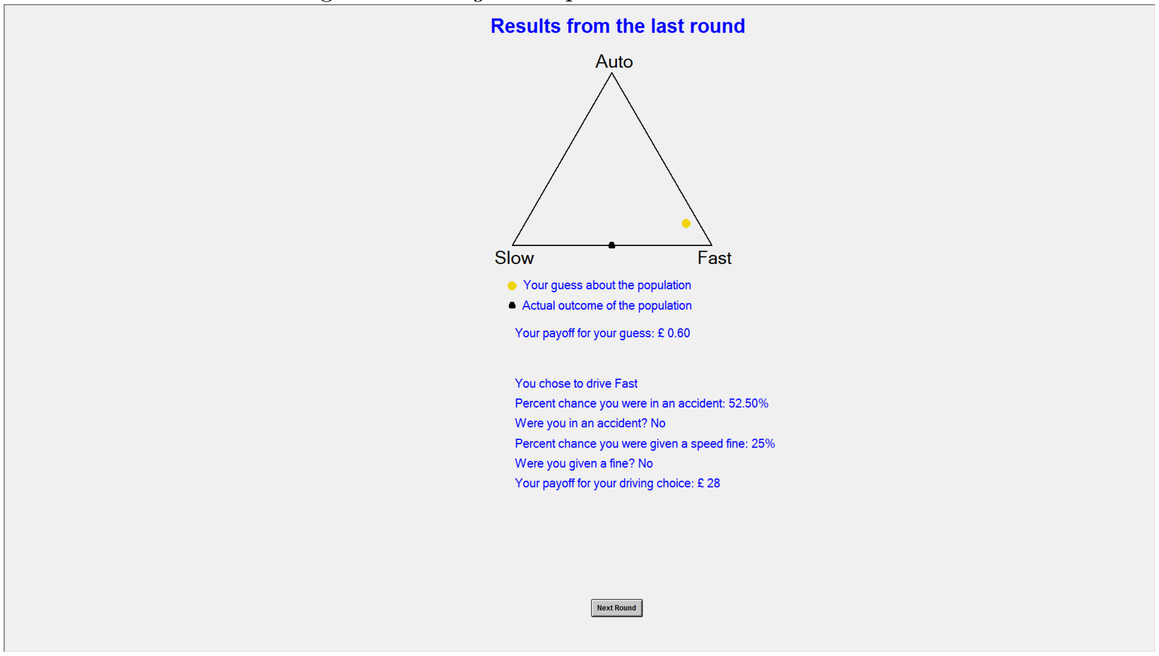

Figure 11: *Endogenous* punishment results screen

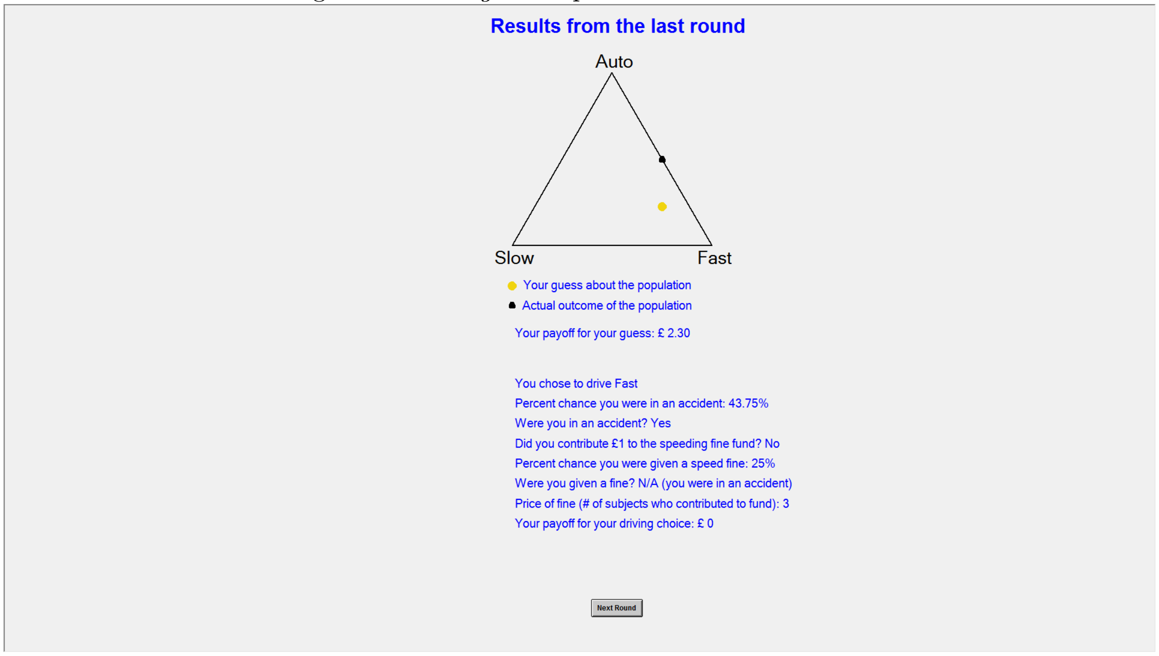

Supplement: S1 File — (PDF) [file pone.0275383.s001.pdf]
