# Supplementary material for: The effectiveness of prosocial policies: Gender differences arising from social norms
Source: PLoS One. 2022 Dec 27;17(12):e0275383. doi: 10.1371/journal.pone.0275383 (PMC9794096; doi:10.1371/journal.pone.0275383)
Supplement: Attachment — Submitted filename: Referee PLOSone.pdf [file pone.0275383.s002.pdf]

Report on PONE-D-22-09104
The effectiveness of prosocial policies: Gender differences arising from social norms
By A Cabrales, R Kendall and A Sanchez

This paper analyses anti-social behavior and the effectiveness of policies aimed at curbing the negative externalities it generates. It does so in the context of driving. The authors present a theoretical model which they use to derive a number of benchmark predictions. Then, they test these hypotheses in the lab. Results show that the policies considered do not have significant aggregate effects but have markedly different results by gender. Male participants respond to these policies by increasing their speed whereas female participants respond in the opposite way. Finally, the authors examine the role of social norms in explaining these results.

Overall assessment

I have refereed this paper for a different journal and I must say that it was shocking to see that the authors had not taken on board the many comments they received from the editor and referees in that submission. This is a paper which tackles an interesting problem. The experimental design is clean. However, the theory section presents a number of problems and it is to a large extent irrelevant; critically, the theory has nothing to do with gender, despite gender is the main topic of the paper.

In addition to the problem with the theoretical model, the paper lacks focus, and feels like a collection of results. The authors remain surprisingly silent about why the policy interventions have the expected effect with females but encounter a backlash with males. This is a puzzle the paper does not tackle. The external validity of the exercise is already limited given the very specific behavior studied (driving) and the subject pool leveraged (university students, some of them surely not drivers). It makes the paper no favor that the authors do not attempt to rationalize this finding.

In addition, the paper is very meandrous. I am sure that there exists a shorter and more compact version of the paper conveying more effectively and clearly the main results of the experiment. But the paper, in its current form, is still a long way from there.

Theoretical model

One caveat of the model is that the authors assume that changing from one speed to another has no effect on the average speed. This can be seen in pp. 7-8 where the average speed of players other than i remains defined as the average speed of the entire population. The equilibrium concept is undefined and there is no explanation given in that regard. The proofs in the appendix show that the concept is likely to be the Nash equilibrium where agents choose the speed that maximize their utility given their beliefs about average speed that must be correct in equilibrium. There is no reason to avoid defining the equilibrium concept as the correctness of the theoretical results hinges critically on that; they cannot be correct or incorrect in a "theoretical vacuum". There are other issues related to the presentation of the model such as that the parameter a_i appearing before being properly introduced; that the utility functions in p. 8 could be presented in a compact way using D_i; that there is no proof of Proposition 3 or at least a formal proof that a reduction in the payoff of F and AS is monotonic in fines and punishments; or that the proof or Proposition 1 assumes $N = 10$ without that being mentioned in the main text (the reader discovers that only when checking the proofs).

Whilst these issues are just a nuisance, it is much more problematic that the main proposition, Proposition 1, and at least one of the hypotheses derived from it, Hypothesis 2, are wrong. These is a serious claim, so let me be very precise now.

Let me start with part 2 in Proposition 1. There it is said that there is no equilibrium where drivers only choose A or only F if the distribution of risk attitudes $G(.)$ has positive mass everywhere in its support $(0, 1)$. The proof in pp. 39-40 claims to prove that assertion by contradiction by showing that for an agent to choose F when everybody else chooses F it must be that $\gamma_i \geq 0.86848$. Well, that is no contradiction. An equilibrium where every agent chooses F can happen with positive probability, actually with probability $(1 - G(0.86848))^N$ if we assume risk parameters are i.i.d. draws from $G(.)$. If all the draws are above that threshold, the all-Fast equilibrium exists. I have gone over this many times and I cannot see why the authors can state that the fact that $\gamma_i \geq 0.86848$ invalidates this. Choosing F when everybody else chooses F does not need to be a best response for all γ_i for that to be an equilibrium. It is enough that it is a best response for all agents and there is a positive probability of that happening (the probability of the γ_i of all players being above that threshold). It can be argued that such equilibrium is unlikely to happen (although that depends on the specific shape of $G(.)$) but what is clear is that Hypothesis 2, which is derived from

this part of the proposition is wrong too; the theory does not lend support to the prediction that "participants in the experiment will never completely coordinate on choosing F". They may, with some positive probability.

Unfortunately, part 1 of Proposition 1 is incorrect as well. The proof is not particularly helpful so consider instead the graph below. The black line corresponds to the locus in the parameter space ($\gamma_i \in (0,1)$ is the risk attitude parameter, $AS \in [0.5,2]$ is the belief about the average speed) where the utility of an agent driving slow is equal to the utility of that agent if they drove fast, that is

$$EU(F) = 2^{\gamma}(1 - 0.35(\frac{9}{10}AS + \frac{2}{10})) = 1 - 0.3(\frac{9}{10}AS + \frac{1}{10}) = EU(S)$$

Parameters are such that $EU(F) < EU(S)$ below that curve. Similarly, in the area below the red curve it holds that

$$EU(A) = 0.5^{\gamma} < 1 - 0.3(\frac{9}{10}AS + \frac{1}{10}) = EU(S).$$

This demonstrates that the statement 1 in Proposition 1 ("There are no beliefs about AS and no value of γ_i for which it is optimal to choose S") is false, as it is a best response to choose Slow for an agent whose γ_i and beliefs of AS are in the area below the black curve. Consider for instance the case of an agent who expects everybody else to play F, i.e. $AS = 2$; if the agent is sufficiently risk averse, i.e., $\gamma_i < 0.5$, their best response is indeed to play S. The intuition is clear and consistent with the strategic substitutes nature of the game.

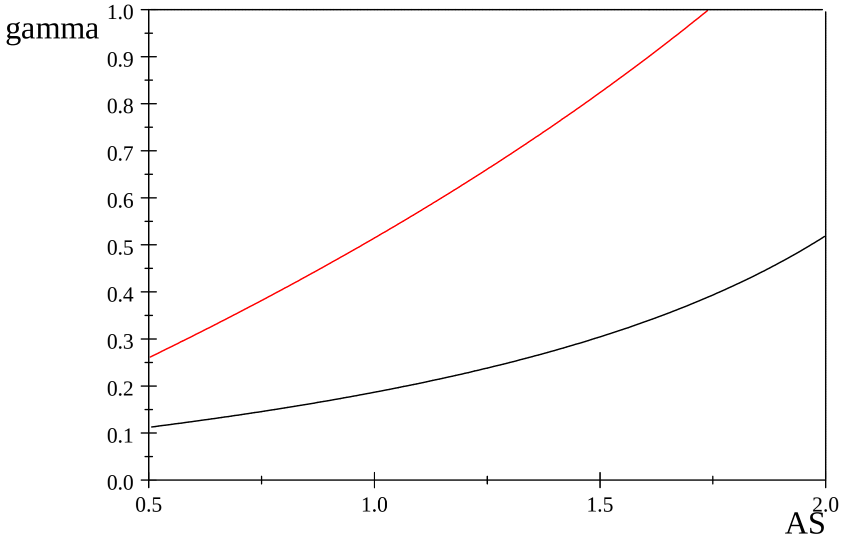

All this forces me to conclude that the model should be scrapped from the paper. It adds very little, it is technically unsound and the way it models social norms is unhelpful in understanding the experimental findings.

Presentation of the results

The presentation of the experimental results is plodding. I already mentioned in my previous report that the analysis of earnings was given too much prominence and could be scrapped. The authors kept it. And whilst I can see that as a matter of taste, I still believe that the current version hosts a proliferation of tables and results (11 tables in the main text, 7 questions and 7 results to a total of 34 pages excluding references). This detracts the paper from making its message come across effectively.

As mentioned above, the writing highlights how the behavior and beliefs of females is affected by the policies implemented. That is not the surprising result. After all, females behave as expected, they abide. They are affected by the policies and change their beliefs. It is the behavior of males what is surprising. They not only do not reduce their speed but increase it. They do not adjust their beliefs. In sum, the results suggest strongly that the interventions emboldened males. This goes against all what should be expected and should, in my view, be highlighted much more. There is some material added about this compared to the last version I saw (in Section 5 and the conclusion), but it is featured only marginally (the main body of the paper remains intact) when it should be central.

Again, I believe that there is a better, shorter (and model free) version of the paper which is shorter and highlights its two main results: 1) Interventions do not reduce AS; 2) the reason is that whilst females respond as expected to the new policies, males are emboldened.

Let me also mention that I am not entirely convinced that the data supports Result 5. The interaction $Framing$x$Female$ in Table 10 is not significant. The coefficients suggest rather that $Framing$ has an effect but that is not gender-specific.

Minor comments

- There are several citations missing (e.g. p.1) and figures not correctly referenced (e.g. p. 15).
- There is no mention to when the experiment was run.
- That the Holt and Laury method was used to elicit risk preferences would probably be better placed in p. 11-12 when describing the experimental procedures.
- Table 14 shows that participants in the Exogenous treatment had different risk preferences than the rest. Could that explain that gender differences in speed are only weakly significant under that policy? (in contrast with the other policies, where gender differences are much stronger).
- It would be nice to see a comment on why *Endogenous* is the treatment that generates the lightest sway in beliefs.
- It would be useful for the reader to have a footnote explaining how guesses of the driving distributions translated into earnings (pp. 11-12).
- Why the regressions on driving choices (Table 4) do not include the previous' round decision as a control?
- Why is the proposed designed a "mixed-agency" scenario? (p.. 33). This concept is unexplained.

- The authors have not bothered correcting the typos already pointed out in submission this referee already evaluated: p. 4, duplicated "However"; p. 4 "being" instead of "been"; p. 10, "Endogeno" instead of "Endogenous"; p. 20, a "to" is missing after "likely"; p. 25, "par ticipants"; p. 30, a "to" is missing after "likely".
