# Supplementary material for: The effectiveness of prosocial policies: Gender differences arising from social norms
Source: PLoS One. 2022 Dec 27;17(12):e0275383. doi: 10.1371/journal.pone.0275383 (PMC9794096; doi:10.1371/journal.pone.0275383)
Supplement: Attachment — Submitted filename: Reply PLoS One.pdf [file pone.0275383.s004.pdf]

GRUPO INTERDISCIPLINAR DE SISTEMAS COMPLEJOS
DEPARTAMENTO DE MATEMÁTICAS
UNIVERSIDAD CARLOS III DE MADRID
Avda. Universidad, 30 – 28911 Leganés, MadrId
http://www.anxosanchez.eu / anxo@math.uc3m.es

uc3m
GISC

Answers to Reviewer 1 and summary of changes made in response to his/her comments

1. This is an interesting paper that sheds light on how different policies can have differential gender effects. It also adds to the long literature on gender effects. The experiments are well designed (with a small caveat-see below) and the results are clear. However, there are lots of typos and spelling mistakes in the entire document.

   We thank the Reviewer for his/her praise of our work and we sincerely apologize for the typos and mistakes. In this new version we have tried our best to fix all those issues.

2. Recent work has shown that providing information on accidents is distracting and actually increases them due to cognitive fatigue (How safe are safety messages?, Gerald Ullman and Susan Chrysler, Science, 21 Apr 2022, Vol 376, Issue 6591, pp. 347-348, DOI: 10.1126/science.abq1757). Your paper is interesting in this aspect as it can be argued that it shows that ex-ante primes (not while at the task) may be a better solution as it does not add to the cognitive fatigue while driving.

   We thank the Reviewer for this comment, which seems very interesting to us. Therefore, we have added in the introduction a sentence about this, thanking the Reviewer in a footnote.

3. Is there work that has looked at differential gender effects on norms? If not, it makes the paper even more relevant.

   This is indeed an important question which we have addressed in the revised version of the manuscript. We now give some examples of that literature in the review, noting explicitly in the new text "the main difference in our case is the context, on the one hand. More important, perhaps, is the fact that in our case males not just comply less, they take advantage of compliers to behave worse."

4. The introduction is not clear and reads confused. It will be a good idea to narrow down what you are doing and writer a clearer introduction.

   We have significantly reduced and focused the introduction and thereby, we hope, made it clearer.

5. Para 2, line 1. Missing citation. Figure numbers appear as ?? later on in the paper too.

   We do not know what is the reason, but we have not been able to nail down this problem because we do not observe any of this in our manuscript. Perhaps it was a product of the compilation of the file in the journal server? In any event, in this new version we are sending a pdf file making sure that there are no missing references.

6. Intro para 2, line 5. Do you mean that the context is preferences over electric vs hybrid (which pollute more than electric)? Again, what is the context dependent element here? I found this confusing to read.

   We are sorry this was not clear in the previous version. We now talk about antisocial behaviors, which we now define in the first paragraph, explaining that these are examples of antisocial behaviors that share features with the driving example.

7. Intro para 2, line 8. I do not see the continuation of the argument here. You are going from social norms that are context dependent to laboratory experiments. There is no relation. These are two separate arguments.

   We agree with the Reviewer on this point. We now make this clearer by separating in different paragraphs and slightly changing the explanation.

8. Para 3. There is a substantial information on driving, by age, gender, highways and country roads. It would be good if you put your motivation in that context. A quick google search told me that speeding only accounts for 28% of the accidents. I think what you are doing is more general than that. Your framework can apply to many scenarios. Maybe thinking about other scenarios where this may apply will broaden the appeal of the paper. A discussion to this regard would be useful.

   We are thankful to the Reviewer for this useful insight. We now mention driver distraction and intoxication as behaviors that cause similar problems and for similar reasons.

9. Last para, page 2. ….such AS higher deaths from both…

   We have corrected this.

10. I find sentences in the introduction that are unrelated to the previous arguments, line 2, page 3, intro is an example.

    In order to clarify what we mean here, we have made the explanation about autonomous vehicles a separate paragraph and tried to explain better that they will make antisocial behavior more "profitable" and thus more worrisome.

11. Page 3, line 8 (is it Manual or Slow?)

    We now clarify by introducing the words "Non Auto".

12. Page 3, line 9, you mention the strategies without having defined them.

    We are sorry about this. We have taken care of this issue by defining strategies before talking about them.

13. You assume that speeding also increases the probability of accidents. This is a ceteris paribus argument. Some clarification on this would be useful.

    We hope that the new explanation about this is clearer and more useful for the reader.

14. Page-4, line 7, "However" repeats.

    We have removed the first instance, which indeed was not necessary.

15. Page 4, line 11, Can start with a new paragraph. It's easier to read the texts if each para starts with a new argument. Page-4 is a long read. It can have new paras at line 11, 19,

We agree with the Reviewer that the previous version was hard to read, so we have included more paragraph breaks.

16. Same page, assesS (spelling).

Corrected.

17. Page-5, first para, last line. I found it hard to understand the sentence.

We now explain that "Indeed, many previous papers show that people internalize social norms more strongly when they come from the community than when they are imposed by an external authority."

18. In the "Relevant literature" section you can include articles on Transport research. There is a substantial literature on driver behaviors. It would be good to have a summary about what we already know. For example, see, "The impact of road advertising signs on driver behavior and implications for road safety: A critical systematic review," Transportation Research Part A: Policy and Practice, Volume 122, April 2019, Pages 85-98.

We thank the Reviewer for pointing out to us this literature. We now discuss it int the following terms: "Given that our experiment has a specific frame we should note there is an extensive literature in transportation science that identifies the effect of gender on driving behavior. Oviedo-Trespalacios et al. (2019) shows that males are distracted more often by roadside signs, but females look at them longer. Jiang (2020) find that women are less aggressive when encountering a yellow light. Interestingly for our research, Jorgensson and Polak (1993) find that although males drive faster in the absence of speeding fines, there is no gender difference when fines are present. More recent research by Elias (2018) also finds no difference in gender attitudes to speeding fines. Notice that in both cases there are no strategic interactions, in the sense that the authors do not consider environments where less speeding by one group makes it easier for others to speed."

19. Para 2, section 2, is your paper about cooperation?

It is more about behavior in social dilemmas, which we now make clearer, hopefully.

20. Page 6, Section2, para 2, line 4. Extra space before parenthesis. This repeats in several places.

We have carried out a global "search and replace" to deal with this issue.

21. Page 5, beginning, you state that, "Because this literature provides ambiguous messages about the effect of gender, we create a model in Section 3 that does not directly account for gender differences. Instead we allow the data to clarify the direction of effects (Section 5)." Then what is the usefulness of your model?

We thank the Reviewer for asking this question because it helps us make our point better. Our model is useful in the sense that we want to detect "anomalous" behavior according to some objective benchmark. As it turns out, just males behave in "unexpected" ways, and now we can point to this.

22. Page 6, para 2: "At this point" is unnecessary. It does not follow from a past argument.

Corrected.

23. Page 7, para 1. Remove "can".

Done.

24. 3.1. The game: a. Explain better how the theoretical models maps into the experimental design. If you want the data to speak for itself, then how relevant is your theoretical framework?

As stated above in our reply to your point 21, we want to detect "anomalous" behavior according to some objective benchmark. As it turns out, just males behave in "unexpected" ways, and now we can point to this. Moreover, we have relegated the theory to an appendix, and just kept the hypotheses that discipline the analysis.

b. "driver" is out of place. Delete.

Done.

c. Define variables in pi.

Done.

d. The accident probabilities are pi. When defining EU on top of page 8, you state that ai defines accident probabilities?

We now make it clearer by saying that ai is a "idiosyncratic factor in accident probabilities".

25. Section 3.2. a. Please clarify what you mean by imperfectly imposed fines. It becomes clearer later on that they are imposed on those that "are not in an accident."

We now clarify that "This only affects participants who were not in an accident, since those in an accident already lose their whole experimental endowment."

b. Endogenous. You have changed two things, the size of the fine which depends on how many vote for it. Better say that it is a variable sign that is increasing in the number of those that vote for the sign. Probably, accounting for beliefs probably controls for this in the analysis. From the results it seems that this should not matter. Discussing this would be useful.

We thank the Reviewer for this pertinent comment. We now clarify that "The probability `of the punishment is the same as in Exogenous but its size but its size depends on how many group members decide to punish the *F* choosing players."

c. Endogenous: "But we social sanctioning…" incomplete sentence.

We now write "Social sanctioning has been shown to support mutual cooperation in large groups."

d. From Proposition 2, can you rank the proportion of players choosing F for each treatment?

While we agree with the Reviewer that this is an interesting suggestion, we do not think we can do that unless we make more assumptions.

e. You define a variable alpha that captures sensitivity to fines. This could capture gender effects too.

The alpha variable is hardwired into the monetary experimental payoffs. There could be additional effects that are akin to those of the monetary alpha, but we do not think we can estimate them structurally with our data.

26. The results are very interesting. Endogenous and framing nudges work stronger for females. It would be interesting to see a discussion on why this is so. For example, brain research has shown that females are more consensus oriented and "aware" of their immediate surroundings (for example read The Female Brain, Louann Brizendine). These results also add to the literature on gender differences in cooperation and competition where they argue that context matters more for females (see, Gender Differences in Preferences, Rachel Croson and Uri Gneezy, Journal of Economic Literature, Vol. 47, No. 2, June 2009, pp. 448-74.

We thank the Reviewer again for considering that our results are interesting. We now have a full paragraph discussing the differential adherence to social norms of the genders in different scenarios, and we also discuss the possible origins, biological or social of those differences. One important difference is, as we note, "in our case males not just comply less, they take advantage of compliers to behave worse."

GRUPO INTERDISCIPLINAR DE SISTEMAS COMPLEJOS
DEPARTAMENTO DE MATEMÁTICAS
UNIVERSIDAD CARLOS III DE MADRID
Avda. Universidad, 30 – 28911 Leganés, Madrld
http://www.anxosanchez.eu / anxo@math.uc3m.es

Answers to Reviewer 2 and summary of changes made in response to his/her comments

Overall assessment

I have refereed this paper for a different journal and I must say that it was shocking to see that the authors had not taken on board the many comments they received from the editor and referees in that submission. This is a paper which tackles an interesting problem. The experimental design is clean. However, the theory section presents a number of problems and it is to a large extent irrelevant; critically, the theory has nothing to do with gender, despite gender is the main topic of the paper.

In addition to the problem with the theoretical model, the paper lacks focus, and feels like a collection of results. The authors remain surprisingly silent about why the policy interventions have the expected effect with females but encounter a backlash with males. This is a puzzle the paper does not tackle. The external validity of the exercise is already limited given the very specific behavior studied (driving) and the subject pool leveraged (university students, some of them surely not drivers). It makes the paper no favor that the authors do not attempt to rationalize this finding.

In addition, the paper is very meandrous. I am sure that there exists a shorter and more compact version of the paper conveying more effectively and clearly the main results of the experiment. But the paper, in its current form, is still a long way from there.

We thank the Reviewer for finding the problem we deal with interesting. We appreciate his/her comments and as we detail below we have tried our best to take them into account. We are sorry if the Reviewer believes we did not do that after receiving his/her feedback in previous occasions. We did try to accommodate all the input we receive although, unfortunately, it is often difficult to conciliate contradictory advice from different Reviewers and Editors. On the other hand, some of the suggestions may be matters of taste and/or factually inaccurate, and those we may not have followed.

Theoretical model

In order to follow as closely as possible the Reviewer's suggestions, we have indeed taken away the model from the core of the paper, leaving just the hypotheses, which as we explain to the other referee are useful because we want to detect "anomalous" behavior according to some objective benchmark. Having said that, we find it important nevertheless to respond to some of the Reviewer's specific comments.

One caveat of the model is that the authors assume that changing from one speed to another has no effect on the average speed. This can be seen in pp. 7-8 where the average speed of players other than i remains defined as the average speed of the entire population.

We respectfully disagree with the Reviewer here as we believe this assertion is not correct. The definition of AS_{-i} clearly states "Speed of the players other than i in the game is given by", which is not the speed of the entire population and it is of course not affected by a change in the speed of i. The speed of the total population is then computed as (N-1)/N AS_{-i} + 1/N S_i, which is what we use throughout the proofs.

The equilibrium concept is undefined and there is no explanation given in that regard. The proofs in the appendix show that the concept is likely to be the Nash equilibrium where agents choose the speed that

GRUPO INTERDISCIPLINAR DE SISTEMAS COMPLEJOS
DEPARTAMENTO DE MATEMÁTICAS
UNIVERSIDAD CARLOS III DE MADRID
Avda. Universidad, 30 – 28911 Leganés, MadrId
http://www.anxosanchez.eu / anxo@math.uc3m.es

maximize their utility given their beliefs about average speed that must be correct in equilibrium. There is no reason to avoid defining the equilibrium concept as the correctness of the theoretical results hinges critically on that; they cannot be correct or incorrect in a "theoretical vacuum".

In this case we would like to apologize to the Reviewer because he/she is correct about this as we had not mentioned this point. In the new version we now clarify: "The risk preferences are the private information of each individual, and the equilibrium concept is Bayes-Nash." In fact, this is a very relevant clarification because the private information is very important in relation to other comment from the Reviewer as we discuss below.

There are other issues related to the presentation of the model such as that the parameter ai appearing before being properly introduced; that the utility functions in p. 8 could be presented in a compact way using Di;

We thank the Reviewer for noting these problems. These two issues have now been taken care of.

That there is no proof of Proposition 3 or at least a formal proof that a reduction in the payoff of F and AS is monotonic in fines and punishments; or that the proof or Proposition 1 assumes N = 10 without that being mentioned in the main text (the reader discovers that only when checking the proofs).

We once again apologize to the Reviewer, because we did forget to add N=10 in the description of the parameters and because we also forgot to include the proof of Proposition 3.

Whilst these issues are just a nuisance, it is much more problematic that the main proposition, Proposition 1, and at least one of the hypotheses derived from it, Hypothesis 2, are wrong. These is a serious claim, so let me be very precise now.

Let me start with part 2 in Proposition 1. There it is said that there is no equilibrium where drivers only choose A or only F if the distribution of risk attitudes G(:) has positive mass everywhere in its support (0; 1): The proof in pp. 39-40 claims to prove that assertion by contradiction by showing that for an agent to choose F when everybody else chooses F it must be that i 0:86848: Well, that is no contradiction. An equilibrium where every agent chooses F can happen with positive probability, actually with probability (1 G(0:86848))N if we assume risk parameters are i.i.d. draws from G(:): If all the draws are above that threshold, the all-Fast equilibrium exists. I have gone over this many times and I cannot see why the authors can state that the fact that i 0:86848 invalidates this. Choosing F when everybody else chooses F does not need to be a best response for all i for that to be an equilibrium. It is enough that it is a best response for all agents and there is a positive probability of that happening (the probability of the i of all players being above that threshold). It can be argued that such equilibrium is unlikely to happen (although that depends on the specific shape of G(:)) but what is clear is that Hypothesis 2, which is derived from this part of the proposition is wrong too; the theory does not lend support to the prediction that "participants in the experiment will never completely coordinate on choosing F". They may, with some positive probability.

In this point, we respectfully disagree with the Reviewer's comment, although we acknowledge that his/her problem here might have arisen from lack of clarity in our writing. As mentioned above, the "The risk preferences are the private information of each individual, and the equilibrium concept is Bayes-Nash." This means that if a (risk-preference) type of a player wants to deviate from the proposed

equilibrium, this is indeed not a Bayes-Nash equilibrium. And that is precisely what we show, there are some types who want to deviate from the equilibria where every type of every player uses A or F.

Unfortunately, part 1 of Proposition 1 is incorrect as well. The proof is not particularly helpful so consider instead the graph below. The black line corresponds to the locus in the parameter space (i 2 (0; 1) is the risk attitude parameter, AS 2 [0:5; 2] is the belief about the average speed) where the utility of an agent driving slow is equal to the utility of that agent if they drove fast, that is EU(F) = 2 (1 0:35(910AS + 210)) = 1 0:3(910AS + 110) = EU(S) Parameters are such that EU(F) < EU(S) below that curve. Similarly, in the area below the red curve it holds that EU(A) = 0:5 < 1 0:3(9 10AS + 110) = EU(S):

This demonstrates that the statement 1 in Proposition 1 ("There are no beliefs about AS and no value of i for which it is optimal to choose S") is false, as it is a best response to choose Slow for an agent whose i and beliefs of AS are in the area below the black curve. Consider for instance the case of an agent who expects everybody else to play F; i.e. AS = 2; if the agent is sufficiently risk averse, i.e., i < 0:5, their best response is indeed to play S: The intuition is clear and consistent with the strategic substitutes nature of the game.

Yet once again we respectfully disagree with the Reviewer, although we have to thank him/her as he/she provides a very nice alternative proof for the result in his/her comment. You claim that point below the red curve have the payoff of S bigger than the payoff of A. But it is easy to see that is incorrect. Take \gamma =1 and AS=0.5. Then it is easy to see that the payoff of A is 1 and that of S is smaller than one. So, in the area above the red curve S > F and in the area below the black curve S > A, but since the two areas have no intersection, our result is in fact true.

All this forces me to conclude that the model should be scrapped from the paper. It adds very little, it is technically unsound and the way it models social norms is unhelpful in understanding the experimental findings.

As can be seen from our previous comments on the theoretical model, we do believe that our theory can be rigorously proven, but of course we agree with the Reviewer that the presentation should have been much better and we thank him/her for helping us making it clearer. In any event, we agree that a shorter paper can be useful and more widely read so we have moved the model to an appendix.

Presentation of the results

The presentation of the experimental results is plodding. I already mentioned in my previous report that the analysis of earnings was given too much prominence and could be scrapped. The authors kept it. And whilst I can see that as a matter of taste, I still believe that the current version hosts a proliferation of tables and results (11 tables in the main text, 7 questions and 7 results to a total of 34 pages excluding references). This detracts the paper from making its message come across effectively.

We have accepted the Reviewer's suggestion and we have moved the analysis of earnings to the Supporting Information file.

As mentioned above, the writing highlights how the behavior and beliefs of females is affected by the policies implemented. That is not the surprising result. After all, females behave as expected, they abide. They are affected by the policies and change their beliefs. It is the behavior of males what is

surprising. They not only do not reduce their speed but increase it. They do not adjust their beliefs. In sum, the results suggest strongly that the interventions emboldened males. This goes against all what should be expected and should, in my view, be highlighted much more. There is some material added about this compared to the last version I saw (in Section 5 and the conclusion), but it is featured only marginally (the main body of the paper remains intact) when it should be central.

We agree with the Reviewer that this is an important finding. In fact, it is a reason why having Hypothesis 3, derived from the theory is useful. We now highlight this result in the introduction, in the results section (both when discussing strategy choice and when discussing social norms) and in the conclusion.

Again, I believe that there is a better, shorter (and model free) version of the paper which is shorter and highlights its two main results: 1) Interventions do not reduce AS; 2) the reason is that whilst females respond as expected to the new policies, males are emboldened.

Let me also mention that I am not entirely convinced that the data supports Result 5. The interaction FramingxFemale in Table 10 is not significant. The coefficients suggest rather that Framing has an effect but that is not gender-specific.

In order to take this suggestion from the Reviewer into account, we now moderate the statement of Result 5 "In the presence of the $Endogenous$ policy condition, female participants are more likely to state Auto as their personal normative belief instead of Fast."

Minor comments

1. There are several citations missing (e.g. p.1) and figures not correctly referenced (e.g. p. 15).

As we answered to Reviewer 1, we do not know what is the reason, but we have not been able to nail down this problem because we do not observe any of this in our manuscript. Perhaps it was a product of the compilation of the file in the journal server? In any event, for this new version we are sending a pdf file making sure that there are no missing references.

2. There is no mention to when the experiment was run.

We are sorry we did not include this information in the previous version of the paper. We now explain that "Experiments were conducted between January and May of 2018 at University College London's Experimental Laboratory for Finance and Economics."

3. That the Holt and Laury method was used to elicit risk preferences would probably be better placed in p. 11-12 when describing the experimental procedures.

Done.

4. Table 14 shows that participants in the Exogenous treatment had different risk preferences than the rest. Could that explain that gender differences in speed are only weakly significant under that policy? (in contrast with the other policies, where gender differences are much stronger).

GRUPO INTERDISCIPLINAR DE SISTEMAS COMPLEJOS
DEPARTAMENTO DE MATEMÁTICAS
UNIVERSIDAD CARLOS III DE MADRID
Avda. Universidad, 30 – 28911 Leganés, MadrId
http://www.anxosanchez.eu / anxo@math.uc3m.es

We thank the Reviewer for the suggestion. We now say "Note, though, that the subject pool is a bit more risk-averse on average (although the difference only has a p-value of 0.06). This could perhaps partially explain the dampened results in Exogenous."

5. It would be nice to see a comment on why Endogenous is the treatment that generates the lightest sway in beliefs.

We are afraid that we are not entirely sure what the Reviewer means here, and therefore we have taken no action. Endogenous is in fact the treatment which generates the largest change in beliefs, especially for females. Perhaps we misunderstand the question?

6. It would be useful for the reader to have a footnote explaining how guesses of the driving distributions translated into earnings (pp. 11-12).

In regard to this question, we would like to point out that this was already stated in the paper in p. 12 of the original manuscript, and we have kept the sentence in the new one. The sentence is: "The amount a participant earned from their guess was 5 minus the difference between their guessed distribution of driving styles and the actual distribution of driving styles in that round. A perfect guess earned 5 and a very inaccurate guess earned 0."

7. Why the regressions on driving choices (Table 4) do not include the previous' round decision as a control?

About this suggestion, we have to say that in an earlier version of the manuscript we had included this variable in the regressions, but in the reviewing process another Reviewer asked us to remove that variable, and we have left it like that.

8. Why is the proposed designed a "mixed-agency" scenario? (p.. 33). This concept is unexplained.

We have removed this unexplained concept and its corresponding sentence from the manuscript.

9. The authors have not bothered correcting the typos already pointed out in submission this referee already evaluated: p. 4, duplicated "However"; p. 4 "being" instead of "been"; p. 10, "Endogeno" instead of "Endogenous"; p. 20, a "to" is missing after "likely"; p. 25, "participants"; p. 30, a "to" is missing after "likely".

We apologize to the Reviewer for this oversight. These are now corrected, with the exception of p. 25, "participants", which we have not been able to find.
