# Supplementary material for: The effectiveness of prosocial policies: Gender differences arising from social norms
Source: PLoS One. 2022 Dec 27;17(12):e0275383. doi: 10.1371/journal.pone.0275383 (PMC9794096; doi:10.1371/journal.pone.0275383)
Supplement: Attachment — Submitted filename: Revision replies.pdf [file pone.0275383.s005.pdf]

Reviewer #2:

I have read with interest this revised version of the manuscript. My overall assessment is that the revision has gone in the right direction but there is still work to be done. The reason is partially that the authors have not incorporated significant comments made in previous reports.

We thank the Reviewer for his/her appreciation of our new manuscript.

On the negative side, I am again shocked that the authors insist in keeping the model. In their response to this referee they mentioned that "we have moved the model to an appendix". However, a description of the model can still be found in p. 4-5. This is not a matter of taste. Leaving aside its correctness and clarity, the model does not help the reader in any way. It actually detracts from the paper. The model has only one source of individual heterogeneity: risk aversion. This alone cannot explain the gender differences observed in the paper. The model cannot explain either the most interesting result in the paper: that men free-ride on women responding to the policies/incentives provided. In other words, the model sheds no light on why "the outcomes may vary for different groups of the population" (p.4). The predictions of the model are either common-sensical (hypothesis 3) or could be obtained with other models and do not actually hold in the lab. The Endogenous treatment could be implemented in the model but it is not... To be honest, it is beyond me why the authors still want to keep such model despite the arguments provided by several referees, including this one. The current version of the paper is some kind of compromise but in addition to the above it is unsatisfactory because it actually leads to a duplicated presentation of treatments (in p. 5 and p. 7). The experiment and its treatments can be presented without the model. So there is really no excuse for keeping it.

We have now removed completely the model.

Other comments:

There are some other loose ends. In addition, the paper could still improve on the formal side.

- The results driving in the previous literature summarized in p. 3 lines 62-66, seem to run against the ones in the paper. This in itself is no problem. However, the authors describe these results as not relevant to theirs since they are obtained in the absence of strategic interactions. This is not entirely correct. Strategic interactions are always present in driving. Maybe in those works cited strategic interactions are less intense because the population of drivers is larger than in the experiment, but certainly they remain.

We thank the reviewer for making us note this point. In order to be more precise, we now write: "A difference with our framework is that in the latter two cases the authors consider environments where less speeding by one group does not makes it easier for others to speed."

- Another proof that the current compromise in the model is not satisfactory is the derivatives in p.4. What are they for? What do they imply? By the way, the last gamma in the first derivative seems to be missing a subscript.

As the model has been removed, this part has been removed as well.

- The description of the experiment has improved but there is still room for further improvement. There is information still missing on session duration, average earnings, structure of payments (e.g. show up fee), etc.

We now write in the experimental design section: "Each experiment session lasted approximately 90 minutes and none of them lasted no longer than 2 hours. The average payment was 23.14 GBP. The maximum payment was 43.9 and the minimum was 5.1. These numbers include a 5GBP showup fee."

- The endogenous treatment seems the most effective and the one where the strongest differences emerge. It seems surprising that the authors do not exploit it more. For instance, there is no discussion on the contribution decisions. Maybe the belief about how many people contributed to the punishment fund were different between genders. The same with the actual choices. If, as the authors suggest in lines 487-8, "female participants would be more responsive to realizing a violation of their (non-Fast) norm", should not they also contribute more to the punishment fund? Sadly, these possibilities remain unexplored.

Thanks for the suggestion. First, regarding the question whether the belief about other participants' contribution to the punishment fund differed by gender. Unfortunately, we cannot answer it because we do not have those data. We did not ask for the participants' beliefs about others' contributions to the punishment fund. On the other hand, we have looked at the contribution decisions by gender, and we now write just after Result 4 that "One interesting question in this context is whether the gender differences in normative beliefs about driving choices extends to the imposition of fines in the Endogenous policy condition. We did not ask a question about norms in the case of fines, but the actions do not differ very strongly. Female participants contribute to the punishment fund 13.5% of the time, which is higher than male participants (8.6%). However, these differences are based on only 8 sessions of the Endogenous treatment and a logit model which clusters at the session level shows this difference to be insignificant (details in the SI file)." The analysis of the punishment decisions was already in the SI file in the previous version, but we thought it was too detailed for the main text.

- I am not sure the study of decisions after being fined is very convincing. The reason is that females who drive Fast might be different from Males who do the same. Fines are not allocated randomly. Because of that, it is not that surprising that males and females respond differently. If this analysis is to be kept this possibility must be mentioned or proof should be provided that the two sub-samples are comparable in observables.

We have eliminated this analysis.

- Does the result about males in Result 4 suggest that males anticipate that there will be a backlash against the policy?

While we believe that the Reviewer's question is a very interesting one, Result 4 simply says females think the norm will change to Auto under Endogenous and males think it will shift to Auto in Framing. We are not entirely sure that this suggests males anticipate a backlash and therefore we have chosen to leave this point out of the discussion.

- It seems a bit weird to state that "our design also allows us to identify the mechanism driving these differences in behavior" (lines 555-7) after having stated "we cannot make a clear identification of the mediator at work here" (lines 533-534). It seems that the toning down of certain statements has not been applied consistently throughout the paper.

We now say "Our design allows us to suggest possible mechanisms driving these differences in behavior, by using question about personal normative beliefs and empirical and normative expectations to elicit social norm".

Minor comments

- "ex ante primes" (p.2). I'm not sure this is correct English. Priming seems more correct than "primes". In any case, all priming is ex ante (otherwise it would be called nudging or some other thing), so the expression seems redundant.

We now say "which raises the question as to whether priming may be useful."

- Similarly, the expression "the average effect on genders" (e.g. lines 521-2) sounds a bit weird. Something like "the differential effect of policies by gender" seems more correct.

We have adopted the Reviewer's suggestion.

- The "or" in Hypothesis 3 can be safely replaced by an "and"

Done.

- There is often a mix up of present and past tense to describe the experiment (for instance, lines 225-235). The convention is to use the past tense but what consistency is more important; either one or the other.

Done.

- The phrasing "participants who chose Fast had a 25% chance to pay a fine" is not very precise

and a bit weird. "Participants who chose Fast were fined with £4 with a 25% chance each round" seems more precise.

Done.

- Result 3 is mentioned in line 358 as if it had been already stated but it has not.

Result 3 was not needed there, and on the other hand we have merged it with Result 4 as per the Reviewer's suggestions, so we have deleted this mention.

- Probably as a remainder of previous versions, lines 358-360 characterize the choices of females as puzzling when it is actually males' decisions the ones really puzzling.

We have deleted the mention of their choices as puzzling.

- The use of beliefs to explain the results on AS would probably be better placed earlier to explain the importance of the exercise. At the moment, the first paragraphs in the "Empirical expectations" section leave the reader without an explanation of why the exercise is worth doing.

We have moved the sentence "Following Bicchieri (2006), a social norm exists in a group if a majority of members share empirical expectations (beliefs about what most others in the group will do) and normative expectations (beliefs about what most others in the group believe one should do)" to the beginning of the section on empirical expectations.

- I found very little differences between Question 1 & 2 on the one hand and Question 3 & 4 on the other. Maybe they could be integrated in just one question each.

We have done as suggested.

- The verb in the sentence in line 540 is inconsistent: "The origin of that gender differential compliance ARE still underexplained"

This has been corrected.
