# Supplementary material for: The effectiveness of prosocial policies: Gender differences arising from social norms
Source: PLoS One. 2022 Dec 27;17(12):e0275383. doi: 10.1371/journal.pone.0275383 (PMC9794096; doi:10.1371/journal.pone.0275383)
Supplement: Attachment — Submitted filename: response.pdf [file pone.0275383.s006.pdf]

All minor changes indicated by the Reviewer have been implemented
